# CYB5R3 in type II alveolar epithelial cells protects against lung fibrosis by suppressing TGF-**β**1 signaling

**DOI:** 10.1172/jci.insight.161487

**Published:** 2023-03-08

**Authors:** Marta Bueno, Jazmin Calyeca, Timur Khaliullin, Megan P. Miller, Diana Alvarez, Lorena Rosas, Judith Brands, Christian Baker, Amro Nasser, Stephanie Shulkowski, August Mathien, Nneoma Uzoukwu, John Sembrat, Brenton G. Mays, Kaitlin Fiedler, Scott A. Hahn, Sonia R. Salvatore, Francisco J. Schopfer, Mauricio Rojas, Peter Sandner, Adam C. Straub, Ana L. Mora

**Affiliations:** 1 Division of Pulmonary, Allergy, and Critical Care Medicine, University of Pittsburgh School of Medicine, Pittsburgh, Pennsylvania, USA.; 2 Dorothy M. Davis Heart and Lung Research Institute, Division of Pulmonary, Critical Care & Sleep Medicine, Department of Internal Medicine, Wexner Medical Center, The Ohio State University, Columbus, Ohio, USA.; 3 Heart, Lung, Blood, and Vascular Medicine Institute, University of Pittsburgh School of Medicine, Pittsburgh, Pennsylvania, USA.; 4 Aging Institute, Department of Medicine, University of Pittsburgh, Pittsburgh, Pennsylvania, USA.; 5 Department of Pharmacology and Chemical Biology,; 6 Pittsburgh Liver Research Center (PLRC), and; 7 Center for Metabolism and Mitochondrial Medicine (C3M), University of Pittsburgh School of Medicine, Pittsburgh, Pennsylvania, USA.; 8 Bayer Pharmaceuticals Wuppertal, Germany.; 9 Hannover Medical School, Hannover, Germany.

**Keywords:** Pulmonology, Cellular senescence, Fibrosis

## Abstract

Type II alveolar epithelial cell (AECII) redox imbalance contributes to the pathogenesis of idiopathic pulmonary fibrosis (IPF), a deadly disease with limited treatment options. Here, we show that expression of membrane-bound cytochrome B5 reductase 3 (CYB5R3), an enzyme critical for maintaining cellular redox homeostasis and soluble guanylate cyclase (sGC) heme iron redox state, is diminished in IPF AECIIs. Deficiency of CYB5R3 in AECIIs led to sustained activation of the pro-fibrotic factor TGF-β1 and increased susceptibility to lung fibrosis. We further show that CYB5R3 is a critical regulator of ERK1/2 phosphorylation and the sGC/cGMP/protein kinase G axis that modulates activation of the TGF-β1 signaling pathway. We demonstrate that sGC agonists (BAY 41-8543 and BAY 54-6544) are effective in reducing the pulmonary fibrotic outcomes of in vivo deficiency of CYB5R3 in AECIIs. Taken together, these results show that CYB5R3 in AECIIs is required to maintain resilience after lung injury and fibrosis and that therapeutic manipulation of the sGC redox state could provide a basis for treating fibrotic conditions in the lung and beyond.

## Graphical abstract

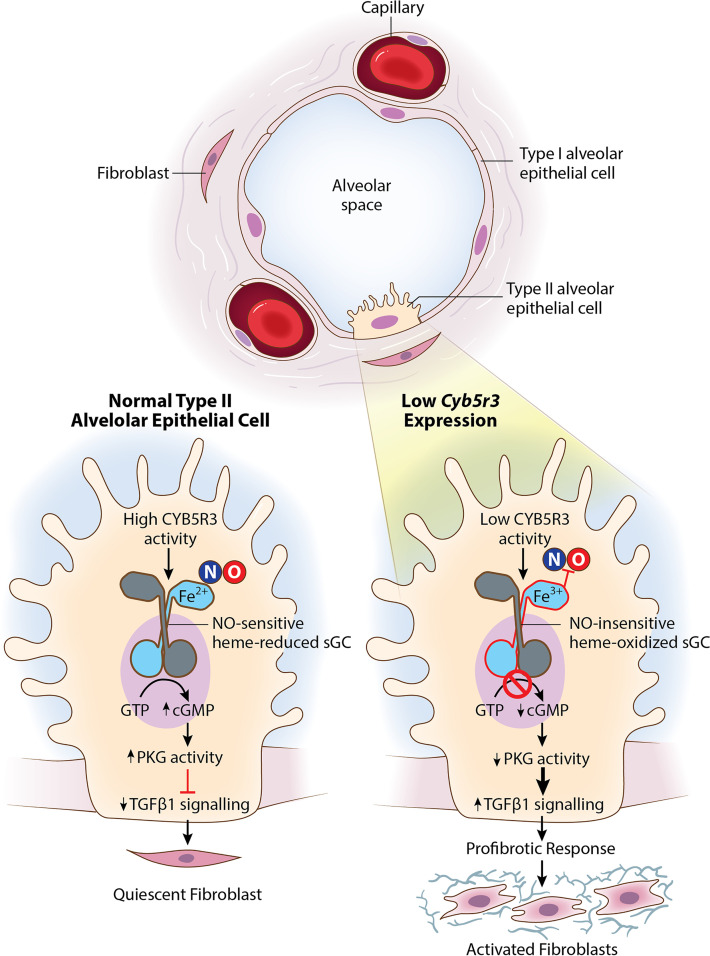

## Introduction

Idiopathic pulmonary fibrosis (IPF) is a chronic, progressive, and deadly disease with few and modest therapeutic options and a median survival of 3–4 years following diagnosis (1, 2). IPF is a disease of age: onset usually occurs at age 50 to 70 years, and most patients are older than 60 at the time of clinical presentation (3). While familial IPF has been linked to disordered telomerase activity and mutations in surfactant proteins, the etiology of many sporadic IPF cases and the mechanisms involved in the aging susceptibility to IPF are largely unknown (4, 5). The most accepted theory in IPF pathogenesis is an epithelial driven process triggered by injury of type II alveolar epithelial cells (AECIIs) that activates pro-fibrotic TGF-β1 signaling (6, 7). TGF-β1 activation leads to altered crosstalk with fibroblasts, myofibroblast activation, and excessive extracellular matrix (ECM) accumulation. While existing FDA-approved therapies slow the physiological progression of IPF, they have adverse effects and do not have an impact on mortality. It is universally appreciated that identification of novel pathways that regulate pro-fibrotic responses and development of the next generation of drugs are needed.

Alterations in reduction and oxidation (redox) homeostasis play a critical role in the pathogenesis of age-related diseases, such as IPF, and have been associated with increased oxidative stress in the lung. Accumulating evidence suggests that elevated expression and activity of NADPH oxidases (NOX enzymes) and mitochondrial dysfunction (8, 9) are critical contributing factors in different cell compartments of the lung. Furthermore, mitochondrial dysfunction (as a hallmark of aging) has been associated with alterations in the NAD^+^/NADH ratio, thus influencing the activity of several downstream proteins, including sirtuins, which can consume NAD^+^ and deplete cells from one of its main sources to restore cellular protection. Recent papers revealed dysfunctional NAD^+^ homeostasis with reduced expression of several NAD^+^ synthesis enzymes in IPF lungs (10, 11). Cytochrome b5 reductase 3 (CYB5R3) is a membrane-bound reductase that uses NADH as substrate and participates in reduction of several key redox molecules (12–14). Recently, we showed that in smooth muscle cells CYB5R3 can directly reduce oxidized heme in soluble guanylate cyclase (sGC), which is required for NO-stimulated 3′5′ cyclic guanosine monophosphate (cGMP) production (12, 15–17). While there is growing evidence for a critical role for CYB5R3 in the cardiovascular system and in aging (18–20), very little is known about the function of CYB5R3 in the context of physiological lung homeostasis and pathology, more specifically during fibrosis.

TGF-β, present in 3 isoforms (TGF-β1, 2, and 3), is a well-recognized pro-fibrotic factor in different organs (21). In the lung, TGF-β is produced by a variety of cell types, including activated alveolar epithelial cells. TGF-β effects in IPF pathogenesis include alveolar epithelial injury, activation of fibroblasts, myofibroblast transdifferentiation, excessive production of ECM, and inhibition of ECM degradation. Although it is known that oxidative stress promotes activation of TGF-β, the precise nature of the mediators regulating this process is not completely understood (8, 22). Considering that increased oxidative stress and redox imbalance in AECIIs are known components of IPF pathogenesis, we sought to investigate if CYB5R3 redox activity was altered in AECIIs from IPF lungs. Here, we demonstrate a potentially novel role for CYB5R3 in AECIIs controlling the TGF-β signaling pathway. In particular, we show that expression of CYB5R3 was diminished in AECIIs of IPF lungs, leading to TGF-β1 signaling activation accompanied by impaired ERK and sGC/cGMP/protein kinase G (PKG) signaling axis. We further show that genetic disruption of CYB5R3 in AECIIs in vivo modulated pro-fibrotic responses and that restoration of dysfunctional sGC/cGMP/PKG signaling by sGC agonists could overcome that. Together, these results establish lung epithelial CYB5R3 as an important regulator of resilience after fibrosis in the lung.

## Results

### CYB5R3 is expressed at lower levels in IPF lungs.

**Figure 1:**
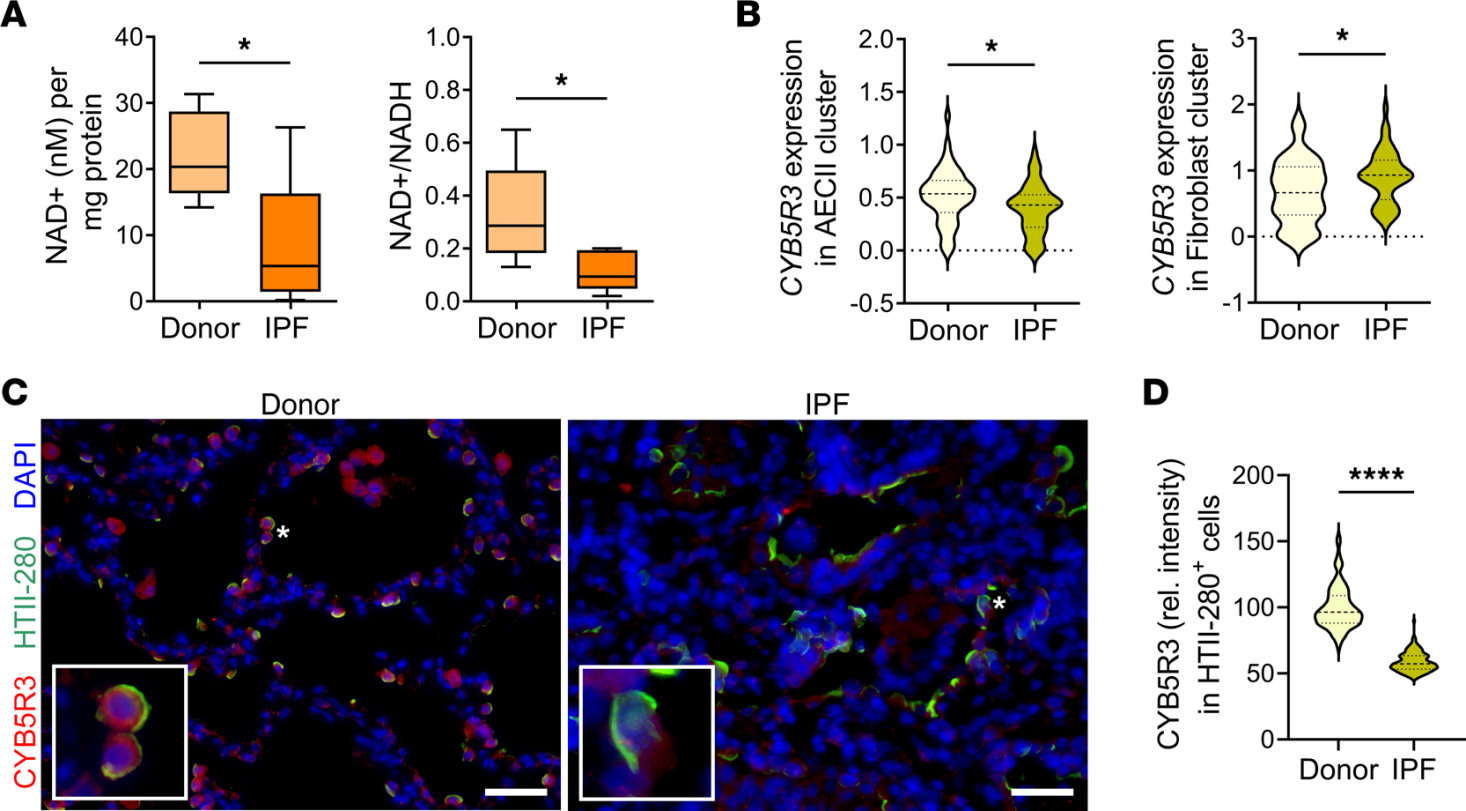
Decreased levels of NAD^+^ in fibrotic lung with reduced expression of CYB5R3 in AECIIs. (**A**) Levels of normalized NAD^+^ and NAD^+^/NADH ratio are reduced in IPF lungs compared with age-matched donors. (Min to max with median, *n* = 5/group.) (**B**) Compilation of mean *CYB5R3* expression in AECII and fibroblast clusters from 3 single-cell RNA-Seq studies comparing donor and IPF lungs. (Violin plots with median and quartiles, *n* = 45 donor/56 IPF.) See Supplemental Figure 1 and ref. 61 for details on data compilation. (**C**) Representative immunofluorescence using anti–HTII-280 (AECII marker, green) and anti-CYB5R3 (red) antibodies, showing reduction in CYB5R3 expression in hyperplastic AECIIs from honeycombs in IPF lung compared with age-matched donors (*n* = 3/group). Scale bars: 50 μm. Representative cells (asterisks) are shown in detail in the insets at ×6.5 original magnification. (**D**) Quantification of CYB5R3 staining (as relative pixel intensity) of individual AECIIs (HTII-280^+^) per condition. (Violin plots with median and quartiles; *n* = 3/group.) See Supplemental Table 1 for demographics of panels **A**, **C**, and **D**. Statistical analysis was performed using 1-way ANOVA with multiple-comparison test (**A**) and unpaired, 2-tailed Student’s *t* test (**B** and **D**); *P < 0.05, ****P < 0.0001.

We have previously reported how changes in the redox homeostasis of the aged lung increase its susceptibility to injury and fibrosis (9, 23). To investigate the relationship between changes in redox coenzymes and lung disease, we determined levels of NAD^+^ and NADH in human total lung lysates. Lungs from patients with IPF showed lower normalized NAD^+^ levels and NAD^+^/NADH ratio compared with control lungs from age-matched donor controls (Figure 1A and Supplemental Figure 1A; supplemental material available online with this article; https://doi.org/10.1172/jci.insight.161487DS1). At the same time, a large data set of mRNA bulk data from the Lung Tissue Research Consortium (Gene Expression Omnibus database accession number GSE47460) (24) did not present significant differences in the expression of NAD^+^-consuming enzymes (Supplemental Figure 1B) but a highly significant decrease in *CYB5R3* expression in IPF when compared with healthy lungs (Supplemental Figure 1C). A compilation of the available single-cell RNA-Seq data analysis revealed a significant reduction of *CYB5R3* mRNA in AECIIs from patients with IPF compared with healthy donors. At the same time, *CYB5R3* transcript levels appeared to be higher in IPF alveolar fibroblasts (Figure 1B and Supplemental Figure 1, D and E). Low protein levels of CYB5R3 in IPF AECIIs were supported by immunofluorescence microscopy (Figure 1, C and D, and Supplemental Figure 1F). In contrast, we were not able to verify a significant change in CYB5R3 expression (mRNA or protein) in primary human lung fibroblasts derived from donor (young and old) lungs when compared with IPF (Supplemental Figure 1, G and H). These data suggest that despite the overall lower levels of NAD^+^/NADH ratio in aged and IPF lungs, the protein expression of CYB5R3 is significantly reduced in the AECIIs of those lungs.

### Deficiency of Cyb5r3 in the AECII increases susceptibility to lung fibrosis.

**Figure 2:**
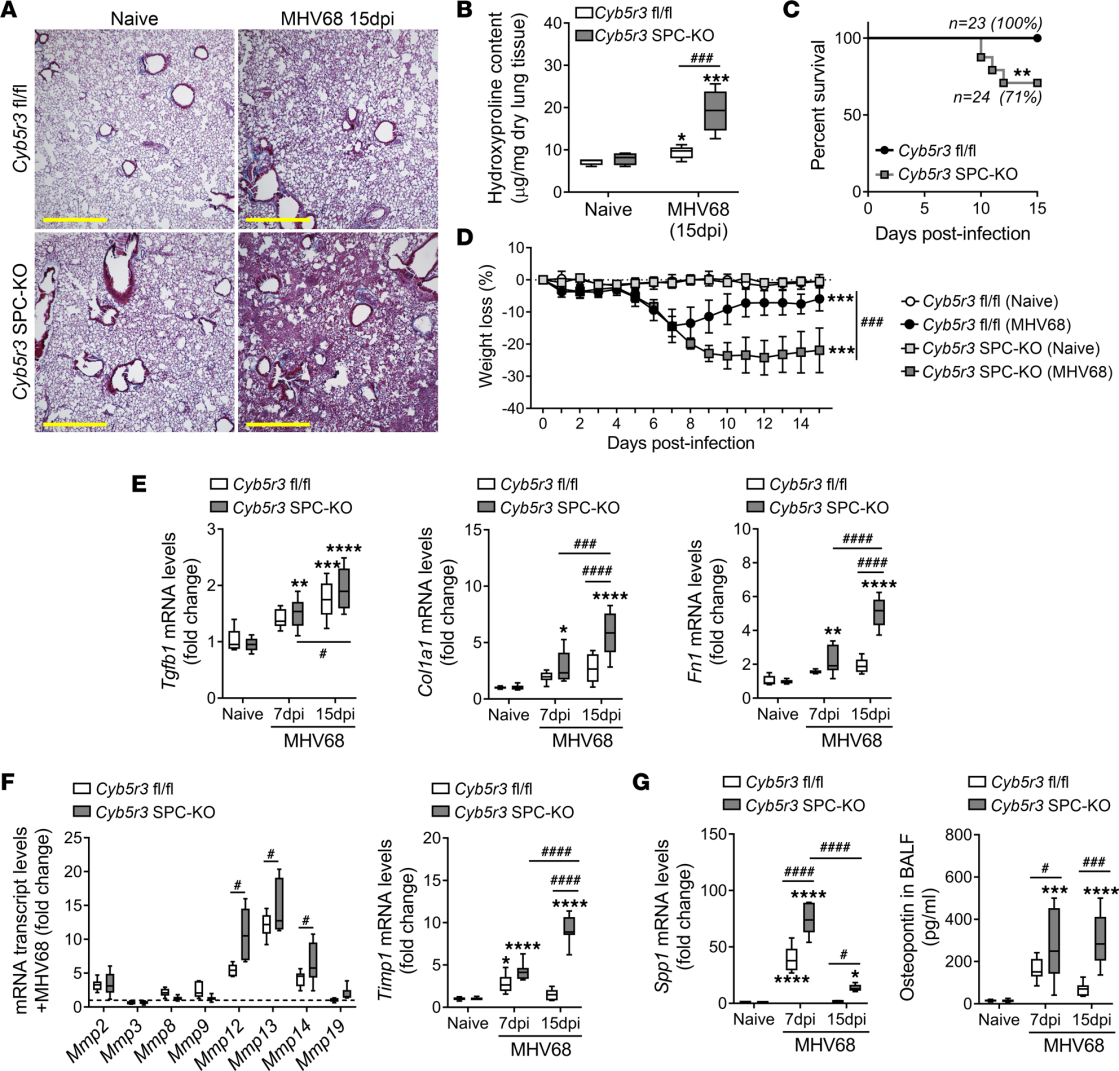
Loss of CYB5R3 in AECIIs increases susceptibility to lung fibrosis after MHV68 infection at day 15. (**A**) Representative Masson’s trichrome staining in lung sections from *Cyb5r3^fl/fl^* and *Cyb5r3* SPC–KO showing increased interstitial collagen deposition (blue) at day 15 after MHV68 infection. Scale bars: 500 μm. (*n* = 6–8/group.) (**B**) Increased collagen deposition in lungs of *Cyb5r3* SPC–KO mice after infection determined by hydroxyproline levels. (Min to max with median, *n* = 5–6/group.) (**C**) Kaplan-Meier survival curve of *Cyb5r3* SPC–KO and *Cyb5r3^fl/fl^* mice after infection (*n* = 23–24, starting mice). (**D**) Weight loss data in *Cyb5r3^fl/fl^* and *Cyb5r3* SPC–KO mice with and without MHV68 infection. More severe weight loss was observed in the infected CYB5R3 AECII–deficient mice. (Data points are mean ± SD, *n* = 6–8.) (**E**) Changes of fibrotic markers *Tgfb1*, *Col1a1*, and *Fn1* mRNA expression levels after virus infection in *Cyb5r3^fl/fl^* and *Cyb5r3* SPC–KO mice. (Min to max with median, *n* = 6- 8/group.) (**F**) Changes in the relative transcript levels of different *Mmp*s and *Timp1* in total lung lysate at 15 days after MHV68 infection. (Min to max with median, *n* = 6–8/group.) (**G**) After infection, *Cyb5r3* SPC–KO mice show higher levels of osteopontin by transcript (*Spp1* mRNA) in total lung lysate and secreted protein in bronchoalveolar lavage fluid. (Min to max with median, *n* = 6–8/group.) Statistical analysis was performed using 2-way ANOVA with multiple-comparison test (**B** and **E**–**G**), Mantel-Cox test (**C**), 1-way repeated measures ANOVA (**D**), and unpaired, 2-tailed Student’s *t* test (**F**), versus naive: *P < 0.05, **P < 0.01, ***P < 0.001, ****P < 0.0001; as indicated: ^#^*P* < 0.05, ^###^*P* < 0.001, ^####^*P* < 0.0001.

To explore the potential role of CYB5R3 in epithelial injury and fibrosis in vivo, we generated an inducible conditional type II lung epithelial cell *Cyb5r3*-knockout mice (*Cyb5r3* SPC–KO), and recombination was confirmed after 2 weeks of doxycycline administration (625 mg/kg) in chow. Recombination was positive in the lung AECII and was not detected in other tissues, such as liver (Supplemental Figure 2). Similarly as in human lungs, we did not observe compensation in expression of CYB5R3 in fibroblasts of *Cyb5r3* SPC–KO mice (Supplemental Figure 2D). We subjected *Cyb5r3^fl/fl^* and *Cyb5r3* SPC–KO mice to our model of lung fibrosis by infection with the murine gammaherpesvirus 68 (MHV68) (Supplemental Figure 3A). We previously showed that MHV68 infection only induces lung fibrosis in susceptible hosts (25, 26). Analysis of lung pathology of *Cyb5r3^fl/fl^* infected mice showed limited interstitial pneumonia and vasculitis while *Cyb5r3* SPC–KO mice developed extended areas of interstitial fibrosis (Figure 2A), which correlated with higher collagen deposition measured by levels of hydroxyproline (Figure 2B). *Cyb5r3^fl/fl^* and *Cyb5r3* SPC–KO showed similar viral loads in the spleen and the lung, indicating that the severity of the fibrosis in the deficient mice was independent of viral persistence (Supplemental Figure 4, A and B). Conditional deletion of CYB5R3 in AECIIs also resulted in increased mortality (Figure 2C), and the surviving mice did not recover their lost weight (Figure 2D). Although there were no significant differences in *Tgfb1* mRNA levels between *Cyb5r3^fl/fl^* and *Cyb5r3* SPC–KO mice, expression of collagen type I (*Col1a1*), and fibronectin (*Fn1*), was increased in the *Cyb5r3* SPC–KO mice at day 15 postinfection (Figure 2E). Higher collagen deposition after infection in *Cyb5r3* SPC–KO mice was accompanied by significantly higher transcript levels of *Mmp12*, *Mmp13*, *Mmp14*, and tissue inhibitor of metalloproteinases 1 (*Timp1*) (Figure 2F) but neither *Timp2* nor *Timp3* (Supplemental Figure 5A). Osteopontin, an important pro-fibrotic glycoprotein located downstream of TGF-β1, was elevated at both total lung mRNA and bronchoalveolar lavage protein levels at day 7 in treated groups, and though almost returned to naive values by day 15 postinfection in Cyb5r3*^fl/fl^* infected mice, the levels remained high in the *Cyb5r3* SPC–KO infected mice (Figure 2G). No other inflammation marker analyzed followed that same pattern (Supplemental Figure 4, C and D, and Supplemental Figure 5, B and C). In addition, no significant changes in *Cyb5r3* expression were found after infection (Supplemental Figure 5D). Finally, evaluation of the redox balance was performed by measuring levels of reduced and oxidized glutathione (GSH and GSSG, respectively) in mouse lungs. We found that the GSH/GSSG ratio was significantly decreased in both infected groups at day 7 postinfection (Supplemental Figure 5E) but only fully recovered in the *Cyb5r3^fl/fl^* group and not in the deficient mice.

**Figure 3:**
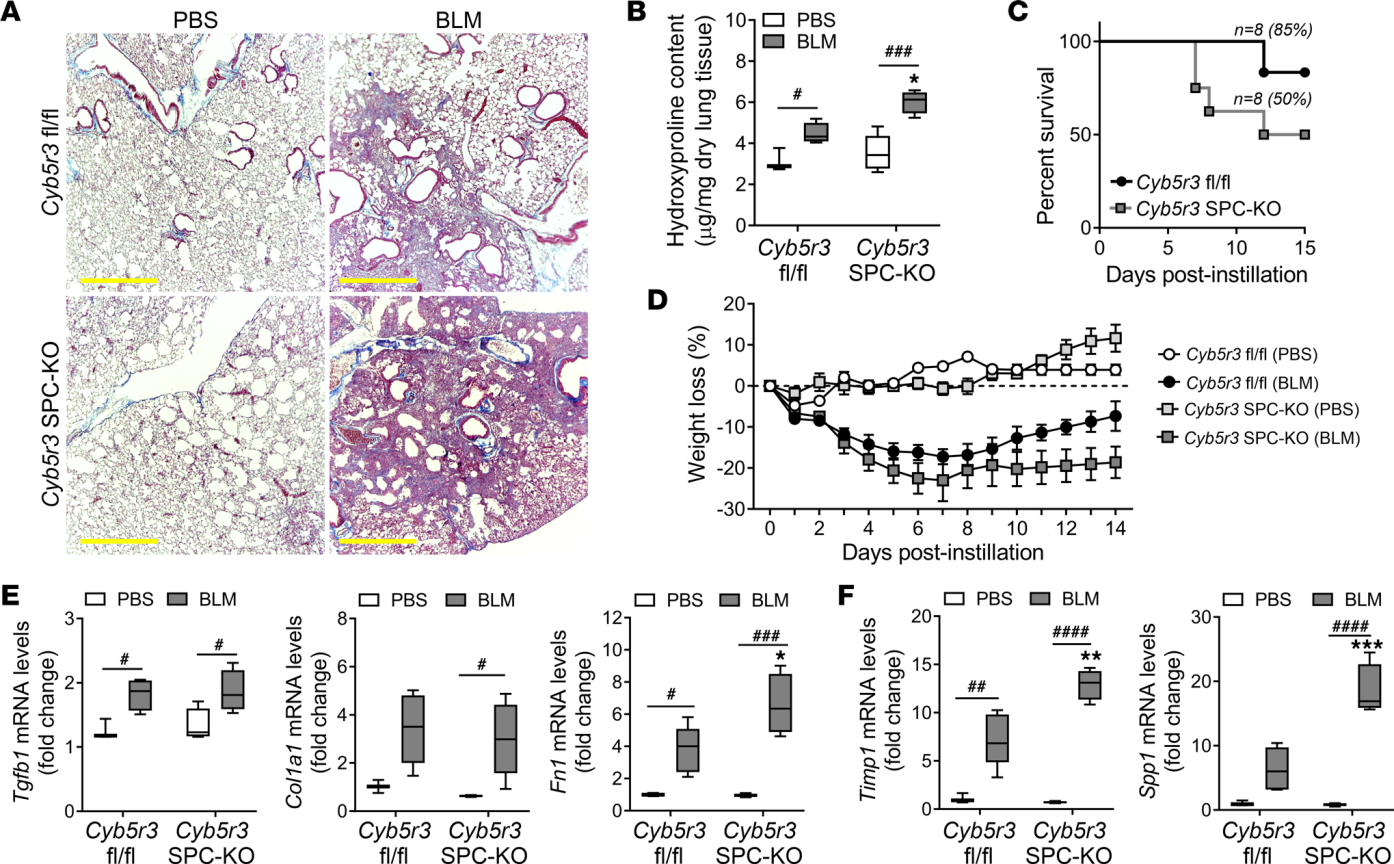
Loss of CYB5R3 in AECIIs increases susceptibility to lung fibrosis after bleomycin with high mortality. (**A**) Representative Masson’s trichrome staining in lung sections from *Cyb5r3^fl/fl^* and *Cyb5r3* SPC–KO at day 14 after bleomycin. Scale bars: 500 μm. (*n* = 4–5/group.) (**B**) Increased collagen deposition in lungs of *Cyb5r3* SPC–KO mice after bleomycin determined by hydroxyproline levels. (Min to max with median, *n* = 4–5/group.) (**C**) Kaplan-Meier survival curve of *Cyb5r3* SPC–KO and *Cyb5r3^fl/fl^* mice after bleomycin (*n* = 8, starting mice). (**D**) Weight loss data in *Cyb5r3^fl/fl^* and *Cyb5r3* SPC–KO mice after bleomycin or PBS treatment. (Data points are mean ± SD, *n* = 4–5.) (**E**) Relative changes of fibrotic markers *Tgfb1*, *Col1a1*, and *Fn1* mRNA levels after bleomycin in *Cyb5r3^fl/fl^* and *Cyb5r3* SPC–KO mice. (Min to max with median, *n* = 4–5/group.) (**F**) Changes in the relative transcript levels of *Timp1* and *Spp1* in total lung lysate at 14 days after bleomycin. (Min to max with median, *n* = 4–5/group.) Statistical analysis was performed using 2-way ANOVA with multiple-comparison test (**B**, **E**, and **F**), Mantel-Cox test (**C**), and 1-way repeated measures ANOVA (**D**), BLM versus PBS: *P < 0.05, **P < 0.01, ***P < 0.001; fl/fl versus SPC-KO: ^#^*P* < 0.05, ^##^*P* < 0.01, ^###^*P* < 0.001, ^####^*P* < 0.0001.

To confirm the susceptibility to lung injury and fibrosis of the *Cyb5r3*-deficient mice, we used a different model of lung injury, the bleomycin-induced lung fibrosis model. *Cyb5r3^fl/fl^* and *Cyb5r3* SPC–KO mice (3 months old) were treated with 1.5 U/kg of bleomycin intratracheally, and their lungs were harvested at day 14 (Supplemental Figure 3A). *Cyb5r3* SPC–KO mice lungs showed extensive fibrosis at day 14 postinstillation, by Masson’s trichrome staining (Figure 3A) and hydroxyproline assay (Figure 3B). Treatment with bleomycin caused higher mortality in the *Cyb5r3*-deficient mice after day 7, reducing the survival to 50% by day 15 with a median survival of only 13 days after bleomycin administration (Figure 3C), as well as considerable weight loss (Figure 3D). In a similar pattern to the MHV68 model, *Tgfb1* mRNA abundance was not increased compared to fl/fl bleomycin-treated animals; however, the expression of fibrosis markers (*Fn1*, *Col1a1*) and TGF-β1 target genes (*Timp1*, *Mmp12*, and *Spp1*) was increased in *Cyb5r3* SPC–KO mice at day 14 postinstillation (Figure 3, E and F, and Supplemental Figure 6, A–C). Bleomycin, as a potent DNA damage agent, caused a significant increase in the expression of senescence-associated cyclin inhibitors even in this short time frame (Supplemental Figure 6D). In both models, after injury, *Tgfb1* levels were not different in the presence or absence of *Cyb5r3* in the AECIIs; however, the response of the TGF-β target genes was increased in the *Cyb5r3* SPC–KO mice. We have assessed the expression of other TGF-β isoforms as well and found the concordant increase in *Tgfb2* and *Tgfb3* in the mouse lungs following bleomycin administration, independently of *Cyb5r3* expression status (Supplemental Figure 6E). For further therapeutic interventions, where longer endpoints needed to be addressed, only the MHV68-induced fibrosis model was used.

Together, these data suggested that CYB5R3 has an important role maintaining AECIIs’ homeostasis and it can be linked to TGF-β signaling that might affect vulnerability to lung injury and development of lung fibrosis.

### Therapeutic interventions aimed at restoring mitochondrial health do not improve virus-induced lung fibrosis in SPC-specific Cyb5r3-KO mice.

**Figure 4:**
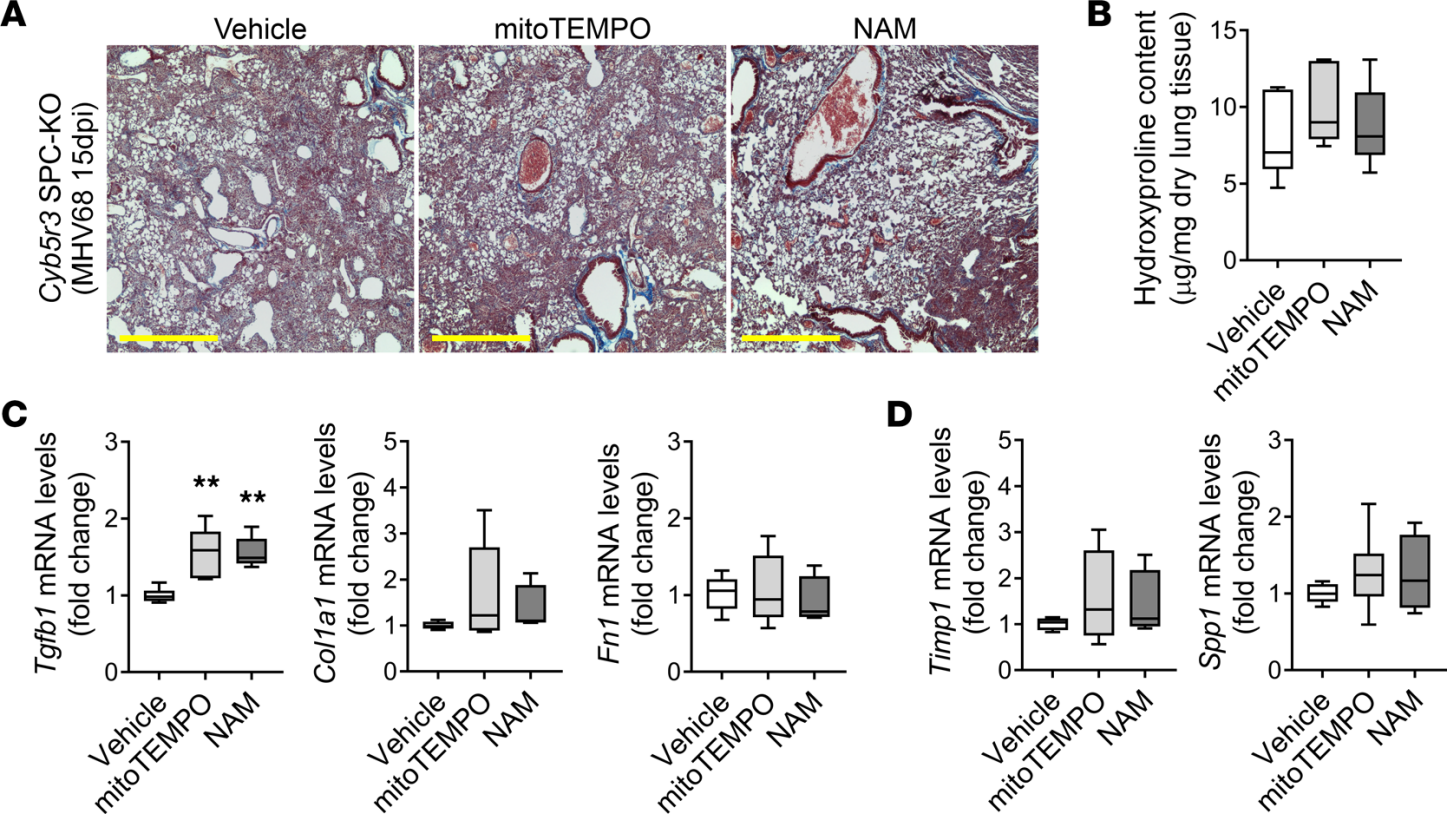
Mitochondrial intervention does not improve virus-induced lung fibrosis in *Cyb5r3* SPC–KO mice. (**A**) Representative Masson’s trichrome staining in lung sections from *Cyb5r3* SPC–KO showing no change in collagen deposition (blue) at day 15 after MHV68 infection after osmotic pump implantation with corresponding treatment (vehicle, mitoTEMPO, NAM) at day 7. Scale bars: 500 μm. (*n* = 5–6/group.) (**B**) Treatment did not change collagen deposition in total lung lysate of *Cyb5r3* SPC–KO mice after infection determined by hydroxyproline levels. (Min to max with median, *n* = 5–6/group.) (**C**) Relative changes of fibrotic markers *Tgfb1*, *Col1a1*, and *Fn1* mRNA expression levels after treatments in *Cyb5r3* SPC–KO MHV68-infected mice. (Min to max with median, *n* = 5–6/group.) (**D**) No detectable change of *Timp1* and *Spp1* transcript levels after treatments in *Cyb5r3* SPC–KO MHV68-infected mice. (Min to max with median, *n* = 5–6/group.) Statistical analysis was performed using 1-way ANOVA with multiple-comparison test (**B**–**D**), versus vehicle: **P < 0.01.

We previously uncovered the critical role of mitochondrial homeostasis in the susceptibility of AECIIs to injury and initiation of paracrine pro-fibrotic signaling (9, 23). Deficiency in CYBR3 in primary fibroblasts has been related to not only NADH accumulation but also to defects in mitochondrial respiration leading to oxidative damage (18). To determine whether susceptibility to lung damage and fibrosis in *Cyb5r3* SPC–KO mice arises due to the mitochondrial ROS buildup or to the imbalance of the NAD^+^/NADH ratio in the alveolar type II cells, we treated MHV68-infected *Cyb5r3* SPC–KO mice with mitoTEMPO or nicotinamide adenine mononucleotide (NAM), respectively, at day 7 postinfection (Supplemental Figure 3B). We evaluated the effectiveness of the systemic therapeutic intervention 7 days later, at 15 days postinfection. There were no differences in weight loss (Supplemental Figure 7, A and B), histopathological features (Figure 4A), and collagen deposition and total collagen content (Figure 4B) between vehicle control and treatment groups. Levels of *Tgfb1* transcript were higher in the intervention groups (Figure 4C). None of the interventions changed the expression of other pro-fibrotic and pro-inflammatory markers tested (Figure 4, C and D, and Supplemental Figure 7, C and D).

There was no mortality registered in both vehicle and treatment cohorts; nevertheless, there was also no benefit in any of these administrations to the infected *Cyb5r3* SPC–KO mice. These data led us to a conclusion that mitochondrion-derived ROS and NAD^+^/NADH imbalance, by themselves, are not the primary outcomes of the CYB5R3 deficiency in AECIIs; therapeutics aimed to improve those outcomes will not succeed in this particular context.

### Therapeutic administration of sGC agonists rescues the fibrotic phenotype in the MHV68 infection mouse model.

We previously showed that CYB5R3 directly reduces oxidized sGC to its biological reduced heme required state (12). In the scenario of deficiency or absence of CYB5R3, this key redox cycling cannot be performed, and the “redox switch” is lost. Interventions aimed directly at increasing sGC activity independently of its redox state (also known as sGC agonists, namely sGC stimulators and sGC activators) could show therapeutic potential under those circumstances. As a direct comparison with the mitochondrial intervention study, we fed MHV68-infected *Cyb5r3* SPC–KO mice sGC activator (BAY 54-6544) or placebo diet, starting at day 7 postinfection, and harvested their lungs at day 15. Despite the short treatment duration and no evident changes in weight loss or *Tgfb1* expression, the CYB5R3-deficient mice on the sGC activator diet after MHV68 infection showed less collagen deposition and decreased expression of pro-fibrotic and pro-senescent markers (Supplemental Figure 8). These data show that the sGC agonist diets could have the potential to improve pro-fibrotic markers in the *Cyb5r3* SPC–KO mice.

**Figure 5:**
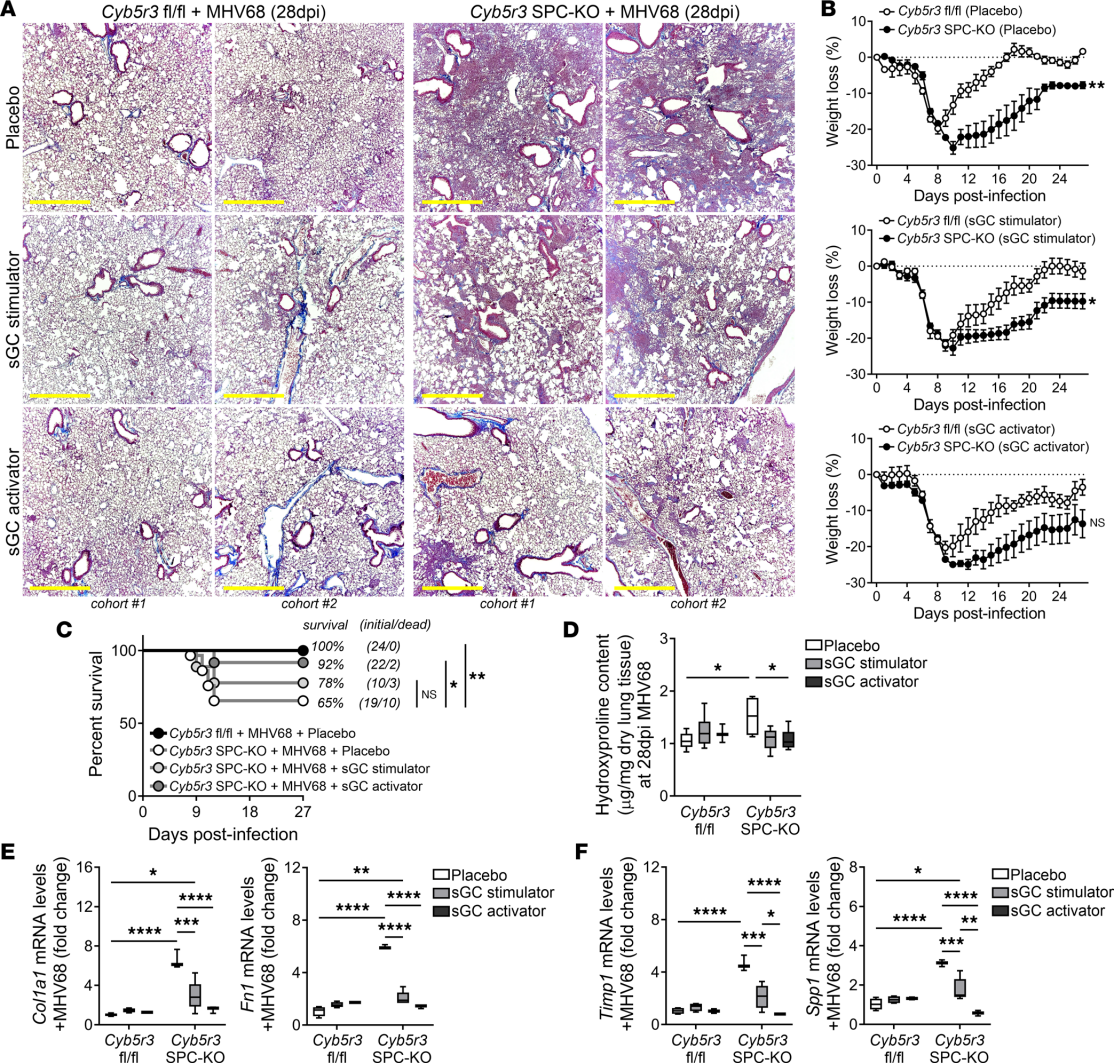
Therapeutic intervention with sGC agonists ameliorates survival and fibrotic outcomes in *Cyb5r3* SPC–KO mice after injury at 28 days. (**A**) Representative Masson’s trichrome staining in lung sections from *Cyb5r3^fl/fl^* and *Cyb5r3* SPC–KO mice at day 28 after MHV68 infection with different interventions representing different experimental cohorts. All interventions were administered mixed in the same base chow composition as the placebo diet and started at day 7 postinfection. Scale bars: 500 μm. (*n* = 4–12/group; see survival details in **C**.) (**B**) Weight loss data comparing fl/fl and AECII CYB5R3–deficient mice after infection in the different treatment arms. (Data points are mean ± SD, *n* = 4–12). (**C**) Kaplan-Meier survival curve of *Cyb5r3* SPC–KO and *Cyb5r3^fl/fl^* infected mice after the treatment with the sGC stimulator (BAY 41-8543) or sGC activator (BAY 54-6544). Initial number of infected mice and final number of deaths per group are annotated beside each survival curve. (*n* = 19–24/group, as a result of multiple cohorts.) (**D**) Collagen deposition in lungs of *Cyb5r3^fl/fl^* and *Cyb5r3* SPC–KO infected mice after the different interventions determined by hydroxyproline levels. (Min to max with median, *n* = 4–12/group.) (**E**) Relative changes of fibrotic markers *Col1a1* and *Fn1* mRNA expression levels after treatments in *Cyb5r3^fl/fl^* and *Cyb5r3* SPC–KO MHV68-infected mice. (Min to max with median, *n* = 4–12/group.) (**F**) Change in transcript levels of *Timp1* and *Spp1* after treatments in *Cyb5r3^fl/fl^* and *Cyb5r3* SPC–KO MHV68-infected mice. (Min to max with median, *n* = 4–12/group.) Statistical analysis was performed using 1-way repeated measures ANOVA (**B**), Mantel-Cox test (**C**), and 2-way ANOVA with multiple-comparison test (**D**–**F**), as indicated: *P < 0.05, **P < 0.01, ***P < 0.001, ****P < 0.0001.

To confirm these pilot results, we investigated the efficiency of the sGC stimulator BAY 41-8543 and the sGC activator BAY 54-6544 in reducing the fibrotic outcomes in *Cyb5r3^fl/fl^* and *Cyb5r3* SPC–KO mice after MHV68 infection (Supplemental Figure 3C). Both WT and KO mice were switched to the different diets in a preventive (3 days prior to infection, Supplemental Figure 9) or therapeutic (day 7 after infection, Figure 5 and Supplemental Figure 10) regimen, then were followed daily for 28 days after infection. Analysis of lung pathology of placebo-fed *Cyb5r3^fl/fl^* infected mice showed limited interstitial pneumonia and vasculitis in contrast to *Cyb5r3* SPC–KO mice that developed extended areas of interstitial fibrosis. *Cyb5r3* SPC–KO mice fed any of the sGC agonist diets showed reduction in collagen deposition by Masson’s trichrome staining (Figure 5A) and hydroxyproline assay (Figure 5D) at 28 days. Therapeutic interventions did not significantly change the ratio of weight loss between the different groups (Figure 5B and Supplemental Figure 11) but had a significant effect on the overall survival of the infected *Cyb5r3* SPC–KO mice (Figure 5C). *Cyb5r3* SPC–KO mice receiving sGC stimulator diet increased their percentage of survival by 13% while in the sGC activator diet group the increase reached 27%, which was also reflected in decreased lung pathology (Figure 5, A and C). As we reported at earlier endpoints, *Tgfb1* mRNA after MHV68 infection showed comparable levels between *Cyb5r3^fl/fl^* and SPC-KO mice with no change at 28 dpi after the intervention with the sGC agonist diets (data not shown). However, expression of fibrotic markers *Fn1* and *Col1a1* (Figure 5E) and TGF-β target genes *Timp1* and *Spp1* (Figure 5F) remained increased in the placebo-fed *Cyb5r3* SPC–KO mice at day 28 postinfection, compared with their fl/fl counterparts. In contrast, expression of those markers was markedly reduced in the lungs of the *Cyb5r3* SPC–KO mice after treatment with sGC stimulator and sGC activator diet (Figure 5, E and F); even the expression of senescence markers was reduced (Supplemental Figure 11C). In all instances, the sGC activator diet led to a higher reduction of all pro-fibrotic markers. Similar results were obtained if the sGC agonist diets were introduced as a preventive treatment (3 days prior to infection), leading to a substantial decrease in the severity of lung damage and fibrosis in the *Cyb5r3* SPC–KO mice after infection (Supplemental Figure 9). Even in the absence of injury, chronic supplementation with sGC stimulator or sGC activator diets decreased the pro-fibrotic milieu of the CYB5R3 AECII-deficient mice (Supplemental Figure 10). Together, these data suggest that, in the absence or deficiency of CYB5R3, interventions aimed at modulating the activity of sGC (independently of its redox state) have a therapeutic potential to ameliorate injury and fibrosis in the lung.

### Deficiency of CYB5R3 in alveolar epithelial cells evokes greater secretion of pro-fibrotic factors upon stimulation with TGF-β1.

**Figure 6:**
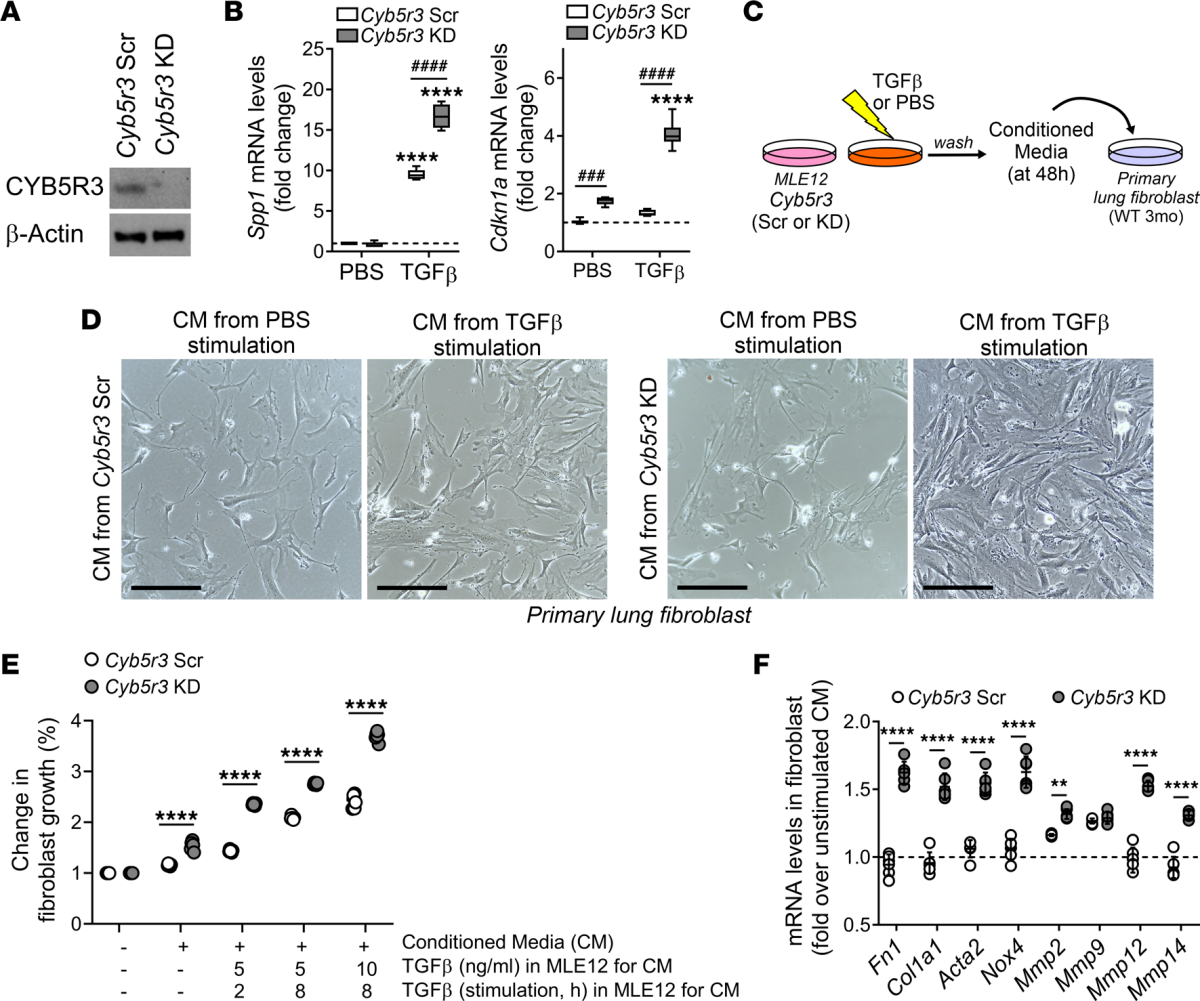
CYB5R3 deficiency in epithelial cell is associated with senescent and pro-fibrotic phenotype. (**A**) Representative immunoblot to confirm KD of CYB5R3 after adenoviral infection of MLE cells. (*n* = 6.) (**B**) Transcript levels of *Spp1* and cyclin-dependent kinase inhibitor 1A (*Cdkn1a*, encoding p21 protein) upon stimulation with TGF-β1, in *Cyb5r3*-Scr and *Cyb5r3*-KD MLE12 cells. (Min to max with median, *n* = 3/group.) (**C**) Schematic of the conditioned media (CM) experiment. (**D**) Representative images of murine primary lung fibroblasts cultured for 24 hours in the presence of CM derived from *Cyb5r3*-Scr or *Cyb5r3*-KD MLE12 cells previously stimulated with PBS or TGF-β1. Scale bars: 200 μm. (*n* = 6/condition.) (**E**) Change in fibroblast growth. Fibroblasts were cultured for 24 hours in the presence of CM derived from *Cyb5r3*-Scr or *Cyb5r3*-KD MLE12 cells previously stimulated with different TGF-β1 treatments compared with primary fibroblasts cultured without CM. For CM generation: TGF-β1 stimulation period was followed by extensive washing; then *Cyb5r3*-Scr or *Cyb5r3*-KD MLE12 cells were cultured with TGF-β1–free media for a total of 48 hours; then CM were harvested. (Individual data with mean ± SEM, *n* = 6/group.) (**F**) Levels of mRNA of different pro-fibrotic markers in total lysate from primary fibroblasts cultured in CM derived from *Cyb5r3*-Scr or *Cyb5r3*-KD MLE12 cells previously stimulated with TGF-β1 (PBS-stimulated CM used as reference state). (Individual data with mean ± SEM, *n* = 6/group.) Statistical analysis was performed using 2-way ANOVA with multiple-comparison test (**B**), PBS versus TGF-β1: ****P < 0.0001; Scr versus KD: ^###^*P* < 0.001, ^####^*P* < 0.0001. Statistical analysis was performed using 2-tailed Student’s *t* test (**E** and **F**): **P < 0.01, ****P < 0.0001.

TGF-β1 is the primary factor that drives fibrosis in different organs (21). In mice, transcript levels of *Tgfb1* after MHV68 infection or bleomycin treatment showed comparable levels between *Cyb5r3^fl/fl^* and *Cyb5r3* SPC–KO mice (Figure 2E and Figure 3E); however, relative expression of TGF-β1 target genes after injury was significantly different in the fl/fl or SPC-KO mice (Figure 2, F and G; Figure 3F; and Supplemental Figure 6C). To investigate how CYB5R3 modulates TGF-β1 downstream signaling, we used MLE12 cells transduced with nontargeted shRNA adenovirus (or scramble, *Cyb5r3*-Scr) or shRNA adenovirus targeting *Cyb5r3* (or knockdown, *Cyb5r3*-KD). Depletion of CYB5R3 was confirmed by immunoblot (Figure 6A). Upon stimulation with TGF-β, activation of classic target genes (such as *Serpine1*) was higher in CYB5R3-deficient cells than in WT counterparts (Supplemental Figure 12). Latent and active TGF-β1 were also evaluated in cell culture media supernatant from MLE12 cells. No differences in secreted active TGF-β1 in cell supernatant were observed between *Cyb5r3*-Scr and *Cyb5r3*-KD cells at baseline (Supplemental Figure 12D). However, after stimulation, *Cyb5r3*-KD MLE12 showed increased total TGF-β1 secretion (data not shown). Analysis of *Spp1* transcript levels showed marked increase after TGF-β1 treatment (9-fold induction) that was further enhanced by depletion of CYB5R3 (17-fold induction) (Figure 6B). Accordingly, conditioned medium from *Cyb5r3*-KD MLE12 cells had a significantly higher amount of secreted osteopontin at baseline, which went up almost 3-fold further upon stimulation with TGF-β1 (Supplemental Figure 12E). Although *Cyb5r3*-KD MLE12 cells did not show any pro-fibrotic phenotype on baseline (data not shown), they exhibited a higher expression of the senescence marker *Cdkn1a*, which was highly increased upon TGF-β1 treatment and not induced in the *Cyb5r3*-Scr cells (Figure 6B).

To investigate if the injured CYB5R3-deficient epithelial cells could propagate this pro-fibrotic and pro-senescent phenotype in vitro, we used conditioned media from TGF-β1–stimulated *Cyb5r3*-Scr and *Cyb5r3*-KD MLE12 cells on mouse primary lung fibroblasts (Figure 6C). Transactivation of fibroblasts only happened in the cultured cells that received conditioned media from stimulated *Cyb5r3*-KD MLE12, compared with the ones that received media from stimulated *Cyb5r3*-Scr MLE12 cells, as seen by direct growth assays (Figure 6, D and E) and by the expression of activation-specific and pro-fibrotic genes (Figure 6F). In conclusion, these data suggest that CYB5R3 deficiency in epithelial cells is associated with a senescent and pro-fibrotic phenotype in vitro.

### Deficiency of CYB5R3 enhances TGF-β1 signaling through an sGC-dependent pathway.

**Figure 7:**
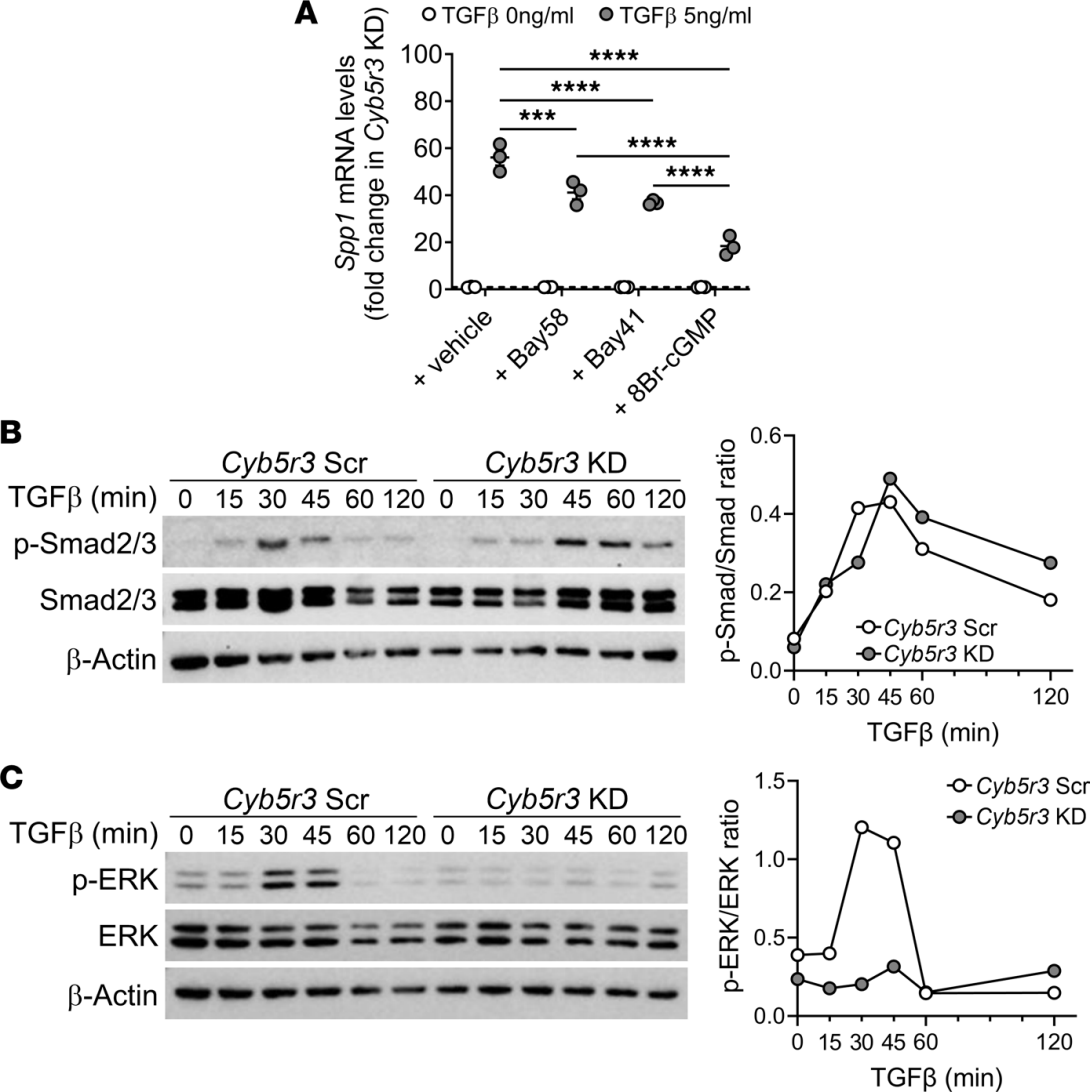
Deficiency in CYB5R3 modifies canonical and noncanonical TGF-β1 pathways. (**A**) Diminution of the TGF-β1–mediated expression of *Spp1* in *Cyb5r3*-KD MLE12 cells stimulated with TGF-β1 in the presence of different sGC agonists. (Individual data with mean ± SEM, *n* = 3/group.) (**B**) Representative immunoblot and quantification of Smad2/3 phosphorylation in *Cyb5r3*-Scr and *Cyb5r3*-KD MLE12 cells stimulated with TGF-β1 at different time points. (Individual data with mean ± SEM, *n* = 3/group.) (**C**) Representative immunoblot and quantification of TGF-β1–dependent ERK phosphorylation in *Cyb5r3*-Scr and *Cyb5r3*-KD MLE12 cells at different time points. (Individual data with mean ± SEM, *n* = 3/group.) Statistical analysis was performed using 1-way ANOVA with multiple-comparison test (**A**), as indicated: ***P < 0.0001; ****P < 0.00001.

Studies in dermal fibroblasts have shown that sGC activation can modulate noncanonical TGF-β1 responses (27). However, whether CYB5R3 participates in the modulation of TGF-β1 signaling is unknown. To investigate the interaction between CYB5R3/sGC/cGMP axis and TGF-β1 signaling, we evaluated *Spp1* transcript levels upon stimulation in MLE12 cells. *Cyb5r3*-KD MLE12 cells in the presence of TGF-β1 had a higher increase in the transcript levels of osteopontin than *Cyb5r3*-Scr MLE12 cells (Figure 6B). However, when the cells were exposed to the stable cGMP analog 8-Bromo-cGMP (8Br-cGMP, 100 μM), sGC activator BAY 58-2667 (Bay58, 1 μM), or sGC stimulator BAY 41-2272 (Bay41, 1 μM), we observed a reduction in TGF-β1–mediated expression of osteopontin (encoded by *Spp1*) (Figure 7A). This effect did not depend on whether TGF-β1 was added as a pretreatment (Supplemental Figure 13, A and C). Treatment with 8Br-cGMP of *Cyb5r3*-KD MLE12 cells also reduced the increased expression of *Cdkn1a* (upon TGF-β1 stimulation) in a dose-dependent manner (Supplemental Figure 13B). To assess the direct effect of the sGC agonists on lung fibroblasts, we cultured primary mouse lung fibroblasts for 24 hours in conditioned media generated from *Cyb5r3*-Scr and *Cyb5r3*-KD MLE12 cells in the presence or absence of Bay41 and Bay58 (Supplemental Figure 14). We found changes neither on growth nor in markers of proliferation and activation because of a direct effect of the sGC agonist on primary fibroblasts (Supplemental Figure 14, A and B). In addition, we did not detect differences in apoptosis analyzed by immunoblot assay of cleaved caspase-3 (Supplemental Figure 14C).

To analyze the canonical TGF-β1 pathway, we followed the Smad2/3 phosphorylation status in TGF-β1–stimulated *Cyb5r3*-Scr and *Cyb5r3*-KD MLE12 cells. Smad2/3 activation peaked within 30 minutes posttreatment in control cells and decreased to baseline levels at 60 minutes. In contrast, in cells depleted of CYB5R3, Smad2/3 phosphorylation peaked later, at 45 minutes, and remained activated for up to 2 hours, suggesting persistent activation of the canonical TGF-β1 signaling pathway (Figure 7B). In parallel, we analyzed the TGF-β1–mediated phosphorylation of ERK1/2. This noncanonical pathway is known to attenuate or modulate downstream TGF-β1–mediated responses (28). We observed ERK phosphorylation peaked at 30 to 45 minutes after TGF-β1 treatment in *Cyb5r3*-Scr MLE12 cells. However, activation of ERK in *Cyb5r3*-KD MLE12 was negligible (Figure 7C). Incubation with sGC agonists or 8Br-cGMP was able to reduce Smad2/3 phosphorylation (Supplemental Figure 14D) while ERK1/2 phosphorylation was rescued by a cGMP analog in response to TGF-β1 (Supplemental Figure 14E).

Together, these data suggest that CYB5R3 modulation of TGF-β1–mediated responses could occur via the sGC/cGMP/PKG axis. These results suggested a synergistic interaction between TGF-β1 signaling and CYB5R3 that modulates expression of TGF-β1–inducible genes.

## Discussion

Although there is evidence that CYB5R3 plays an important role in maintaining cell homeostasis in aging and disease (16, 18–20, 29, 30), very little is known about the function of CYB5R3 in the context of lung physiology and response to injury. Here we showed that deficiency of CYBR3 in AECIIs led to the prolonged activation of the pro-fibrotic factor TGF-β1 and increased susceptibility to lung fibrosis. We used AECII-specific *Cyb5r3*-KO mice and *Cyb5r3* shRNA–KD MLE12 cells to investigate a prospective pathway that modulates TGF-β1 downstream signaling. Through these investigations, we also could demonstrate that sGC agonist interventions had therapeutic benefits in the amelioration of lung fibrosis. Our data show, for the first time to our knowledge, that CYB5R3 indirectly modulated pro-fibrotic signaling in AECIIs, which could be further propagated to a tissue level, involve the other cell types, and ultimately contribute to the remodeling of the lung following injury.

It has been shown that CYB5R3 deficiency has a negative effect on mitochondrial health (31), prompting us to investigate therapeutic interventions including administration of mitoTEMPO (MT) (32) and NAM. The mechanism of action of MT involves scavenging superoxide (33), while NAM is a precursor for NAD^+^, a molecule that serves as a substrate for a number of critical redox reactions, and is formed from NADH via CYB5R3 activity. There appeared to be no obvious benefit in MT or NAM administration to KO animals in terms of histopathology, collagen deposition, or markers of fibrosis at 15 days postinfection. This led us to conclude that mitochondrion-derived ROS and the NAD^+^/NADH ratio alterations do not have a prominent role in CYB5R3 deficiency–related outcomes in the susceptible mouse model.

One of the important heme-containing enzymes that depends on CYB5R3 reductase activity is sGC, the NO receptor (12). During lung development, endothelial NO synthase is highly expressed to promote angiogenesis and lung growth through an NO/sGC-dependent mechanism (34, 35). Inhibition of sGC, on the other hand, has been shown to reduce surfactant protein synthesis (36). Clinical applications of NO or NO-donating drugs are hampered by insufficient availability of bioactive NO, nonspecific interactions, and the risk of oxidative stress and tachyphylaxis. In contrast, the use of sGC agonists can lead to NO-independent and direct stimulation of sGC activity. Currently there are 2 types of sGC agonists; both classes act as sGC agonists and are divided into sGC stimulators, which augment sGC activity without NO and in a synergistic fashion with NO when the heme group resides in the ferrous (Fe^2+^) state, and sGC activators, which increase sGC activity also independent of NO, when the heme iron group is oxidized (Fe^3+^) or deficient. Riociguat, an sGC stimulator, has FDA approval for pulmonary arterial hypertension and chronic thromboembolic pulmonary hypertension (37), and more recently the sGC stimulator vericiguat was approved for chronic heart failure (38). In addition to artery relaxation and the increased blood flow elicited by augmenting cGMP levels, other beneficial effects include increased mitochondria biogenesis and ATP production (39), antiinflammatory actions (40), antifibrotic effects (41), increased antioxidant defense systems (41), and antihypertrophic effects (42). While the potential therapeutic effect of sGC agonists in pulmonary fibrosis has not been examined to our knowledge, mounting evidence in experimental models has shown improved hemodynamics and antifibrotic results. Consistent with our previous work, we show that loss of CYB5R3 enhanced responsiveness to the sGC activator compared with the sGC stimulator. These data are in line with the idea that CYB5R3 protects against sGC oxidation under fibrotic stress conditions to preserve cGMP signaling. Although sGC activator therapy has been shown to be efficacious in our model and in a broad variety of other preclinical disease models, human trials with the sGC activator were halted early because of systemic hypotension (43). However, new oral formulations and dosing regimens of sGC activators that limit the systemic hypotension side effect are in development and might overcome the limitation of first-generation molecules (44, 45).

Mechanistically, a number of studies to date have shown that sGC activation can modulate TGF-β1 responses (reviewed in ref. 46). Studies have shown that the TGF-β1 signaling pathway is a major player in tissue fibrosis, including IPF (47), and this signaling pathway involves several critical phosphorylation events. Upon binding to a cell membrane receptor complex, TGF-β1 activates receptor serine/threonine kinase, which then phosphorylates Smad2/3 factors that, upon binding with Smad4, are capable of nuclear translocation and upregulation of target genes. This canonical pathway is counterbalanced by alternative signaling that includes activation of ERK1/2 and prevention of Smad2/3 nuclear translocation through phosphorylation of its linker site. Previously, inhibitory effects of cGMP on TGF-β1 signaling were observed in vascular smooth muscle cells, attributed to the activity of cGMP-dependent PKG. This resulted in enhanced sequestering of Smad3 by β2-tubulin (48), increased proteosomal degradation of Smad2 (49), and phosphorylation (and activation) of ERK1/2 (50, 51).

Based on that evidence, we hypothesized that AECII CYB5R3-KO mice may be responsive to sGC agonists. Indeed, our results indicated there was a clear benefit as evidenced by a decrease in mortality rate and amelioration of pulmonary fibrosis in bleomycin/MHV68-treated AECII CYB5R3-KO mice that were fed sGC agonist diets. An important observation made during the in vivo experiments was that despite significant upregulation of its target genes, expression of TGF-β1 itself was not significantly different between WT and KO mice after virus or bleomycin administration at various time points. This suggests there is a major inhibitory effect on the TGF-β1 pathway itself in KO mice after sGC dietary modulation. Further investigation revealed that carboxyl terminal Smad2/3 phosphorylation, necessary for downstream signaling, was observed in KD cells for a longer period following exogenous stimulation with TGF-β1. At the same time, there was no evidence of ERK1/2 phosphorylation in KD cells, which might have contributed to the lingering unimpeded TGF-β1 signaling. The consistent reduction in ERK1/2 phosphorylation itself may have additional effects on the cell physiology, including decrease in mitophagy (52) and susceptibility to apoptosis (53). As expected, increased TGF-β1 signaling in KD cells was ameliorated upon treatment with cGMP analogs or sGC agonists. Proximity of the AECII basal membrane to fibroblasts in the interstitium may allow paracrine stimuli from AECIIs to kick-start further propagation of pro-fibrotic signaling. Indeed, this was verified after incubating primary murine lung fibroblasts in the conditioned medium from TGF-β1–stimulated *Cyb5r3*-KD MLE12 cells. We took into account that primary effector cells of fibrosis can also be affected by sGC stimulators and activators (54) but have not observed a significant decrease in proliferation or increase in apoptosis of primary mouse lung fibroblasts following incubation with conditioned medium from TGF-β1–stimulated *Cyb5r3*-KD MLE12 cells and treatment with sGC agonists. On the other hand, this may be the reason why, in our mouse model, sGC stimulators had a beneficial effect as well, though not as great as activators, because it acted on an NO-sensitive sGC that was present in the other cell types.

TGF-β has been shown to induce or accelerate senescence (55, 56). Further, senescence in epithelial cells is also believed to play a role in the fibrogenic process of IPF (57), and CYB5R3-KD cells were shown to exhibit a senescent phenotype in previous studies (18, 20). Concomitantly, we observed an increase in the senescent markers *Cdkn1a* and *Cdkn2a* in the *Cyb5r3* SPC–KO mouse lungs and *Cyb5r3*-KD MLE12 cells, compared with WT controls, further exacerbated by bleomycin/MHV68 challenge or TGF-β1 stimulation. In contrast, sGC dietary modulation corresponded to a decrease in senescent gene expression in mouse lungs. Furthermore, we demonstrated the involvement of the sGC/cGMP/PKG axis by establishing a dose-dependent response of TGF-β1–stimulated *Cyb5r3*-KD MLE12 cells to 8Br-cGMP, resulting in abrogation of the expression of *Spp1* — one of the major TGF-β1 target genes, also implicated in the acquisition of senescent phenotype (58). We also observed the impact of these sGC modulators on the canonical TGF-β1 signaling pathway by registering a decrease in Smad2/3 phosphorylation. Although the effects of sGC modulators on noncanonical ERK1/2-mediated signaling were outside the scope of this study, a cGMP analog (8Br-cGMP) was able to partially rescue ERK1/2 phosphorylation, necessary for the suppression of the initial response to TGF-β1.

Taken together, our data point at the critical role of CYB5R3 in maintaining homeostatic AECII responses during lung injury and repair by modulating TGF-β1 signaling pathway through an sGC/cGMP/PKG axis. CYB5R3 is a key regulator of redox state and during aging might become upregulated associated with the enhanced disruption of redox homeostasis in the cells. In fact, CYB5R3 expression has been reported to increase with age in the lung (59). Studies in cells and mice revealed that overexpression of CYB5R3 may lead to increased protection against oxidative stress and against apoptosis on a cellular level and to general health improvement (29, 60). Deficient expression of CYB5R3 in the IPF lung might impair potential adaptive responses and resilience after injury and fibrosis. New IPF therapies are greatly needed to combat this deadly disease. The results from this study link CYB5R3 deficiency to the upregulation of pro-fibrotic TGF-β1 signaling, suggesting that strategies aiming at CYB5R3 expression and activity, such as the use of sGC agonists, may lessen the severity of pulmonary fibrosis, specifically in the context of the aging lung.

## Methods

### Human lung donors and patients with IPF

Human lung tissue was collected from excess pathologic tissue after lung transplantation and organ donation and from patients with IPF receiving transplanted lungs at University of Pittsburgh Medical Center. Demographic characteristics of the cohorts can be found in Supplemental Table 1. Institutional approval is detailed in “Study approval.”

### NAD+/NADH detection in lung lysates

Total NAD (NADH and NAD^+^) and NADH were measured to determine the NAD^+^/NADH ratio in lung tissue using an NAD/NADH quantitation colorimetric kit (K337-100, BioVision, Inc.) in accordance with manufacturer’s protocol. Briefly, an average of 25 mg of frozen lung tissue was washed in cold PBS, thawed on ice, and homogenized in NADH/NAD extraction buffer. The amount of NAD and NADH was measured in the supernatant, and NAD^+^ was calculated by subtracting NADH from total NAD. Finally, total amount of protein was measured in the remaining lysate sample using Pierce BCA Protein Assay (Thermo Fisher Scientific, 23225)

### Gene expression from bulk mRNA and single-cell RNA from IPF lungs

Publicly available data (available on the NCBI Gene Expression Omnibus [GEO] database) were used to plot gene expression from bulk expression or from specific cell clusters (as defined by the authors of this compilation, ref. 61). The accession codes for the data sets used are GEO GSE47460, GSE128033, GSE135893, and GSE136831. For heatmap generation, data from the set GSE128033 were used, and the *z* scores of the normalized fold-change expression were plotted.

### Immunofluorescence analysis of human lungs

Formalin-fixed, paraffin-embedded lung tissue obtained from patients with IPF (*n* = 3) and age-matched donors (*n* = 3) was cut into 5 μm sections. A total of 3 sections per block were incubated in DAKO antigen retrieval buffer at pH 9.0 (Agilent, S236784-2) for antigen retrieval and then blocked in 10% goat serum in PBS for 1 hour at 4°C. The sections were incubated overnight at 4°C with primary antibodies against HTII-280 (1:200; Terrace Biotech, TB-27AHT2-280) and CYB5R3 (1:100; Proteintech, 10894-1-AP). Secondary antibodies used were Alexa Fluor 488–conjugated goat anti–mouse IgM (μ chain) (1:500; Invitrogen, A-21042) and Cy3-conjugated goat anti–mouse IgG2b (H+L chains) (1:200; Proteintech, SA00009-1). DAPI was used as nuclear counterstain (1:5,000; Invitrogen, D1306). Prior to nuclear counterstain sections were treated with TrueVIEW autofluorescence quenching reagent (Vector Laboratories, SP-8400-15) per manufacturer’s instructions. At least 10 random fields of view per section were photographed (20× objective) on an Axio Observer D1 microscope (ZEISS). Quantification of the CYB5R3 signal in the cells positive for HTII-280 was performed using MIPAR software (62); 88–168 individual AECIIs per lung were measured.

### Generation of the Cyb5r3 SPC–KO mice

Animals were housed in approved USDA OLAW-registered and AAALAC-accredited facilities at the University of Pittsburgh, in ventilated racks with automatic water systems on a 12-hour light/12-hour dark cycle with access to a standard chow (unless fed a specific diet for recombination or treatment). Young (3 months) C57BL/6J mice were purchased from Jackson Laboratory (000664), and the rest of the mouse strains used in this study were generated and bred at the University of Pittsburgh.

Mice with *Cyb5r3* exon 3 coding region flanked with *LoxP* recombination sites (*Cyb5r3^fl/fl^*) were generated by the University of California as part of the Davis Knockout Mouse Project Repository [Cyb5r3^tm1a(KOMP)Wtsi^, CSD29891] and were previously described (15). Mice carrying a doxycycline-inducible Cre recombinase under the control of human surfactant pulmonary-associated protein C (SFTPC) promoter were generated by breeding B6.Cg-Tg(SFTPC-rtTA)5Jaw/J (IMSR_JAX:006235, Jackson Laboratory 006235) and B6.Cg-Tg(tetO-cre)1Jaw/J (IMSR_JAX:006234; Jackson Laboratory 006234), as previously described and validated (63). Then *Cyb5r3^fl/fl^* and mice carrying a doxycycline-inducible Cre under the control of SPC gene regulatory sequence were bred to generate homozygous inducible conditional AECII *Cyb5r3*-KO mice (*Cyb5r3*/tetO-cre/*Sftpc*-rtTA).

All mice used in this study were genotyped by PCR to detect *Cyb5r3^fl/fl^*, tetO-cre, and SFTPC-rtTA sequences as described (15, 63). To promote recombination and generate homozygous conditional AECII *Cyb5r3*-KO mice (*Cyb5r3* SPC–KO), mice were fed doxycycline chow (625 mg/kg, Envigo) for 2 weeks before injury induction.

### Animal injury models

#### MHV68-induced lung fibrosis.

*Cyb5r3^fl/fl^* and *Cyb5r3* SPC–KO mice were inoculated intranasally with saline solution or 5 × 10^4^ PFU of MHV68 as described previously (9) and monitored daily. In the absence of treatment, mice were fed doxycycline chow throughout the duration of the experiment, and lungs were harvested at day 15 postinfection.

#### Bleomycin-induced fibrosis.

A single intratracheal dose of 1.5 U/kg of bleomycin (dissolved in sterile USP-grade PBS) or sterile USP-grade PBS was administered to 3-month-old *Cyb5r3^fl/fl^* and *Cyb5r3* SPC–KO mice. Mice were sacrificed for organ harvest at day 14 postinstillation. Mice were fed doxycycline chow throughout the duration of the bleomycin experiment.

### Animal treatments

#### Mitochondrial intervention studies.

Before the intervention, 3-month-old *Cyb5r3*/tetO-cre/*Sftpc*-rtTA were fed doxycycline chow 14 days to induce recombination and remained on this diet for the duration of the intervention. Then *Cyb5r3* SPC–KO mice were infected with MHV68 (at day 0). At day 7 postinfection, *Cyb5r3* SPC–KO MHV68-infected mice were anesthetized with isoflurane, and osmotic mini-pumps (Model 1002, Alzet) with NAM, MT, or vehicle (injectable saline solution) were inserted subcutaneously between the mice’s shoulders. The interventions were delivered continuously from the pump at a rate of 0.25 μL/h to achieve final doses of 1.5 mg/kg/d of MT (Cayman Chemical, 16621) or 150 mg/kg/d of NAM (MilliporeSigma, N0636). Mice were monitored daily, and lungs were harvested 7 days after intervention (14 days postinfection), frozen, and/or fixed for subsequent analysis.

#### Treatments with the sGC agonists.

Mice were placed on the following experimental diets: base nutritional chow (placebo diet), sGC stimulator (Bay41, 150 ppm) mixed in base nutritional chow (sGC stimulator diet), and sGC activator (Bay54, 80 ppm) mixed in base nutritional chow (sGC activator diet). All diets were manufactured by Sniff Spezialdiäten GmbH and provided by Bayer AG. We previously reported plasma drug levels from these diets’ formulations (44). Before the intervention, 2-month-old *Cyb5r3^fl/fl^* and *Cyb5r3*/tetO-cre/*Sftpc*-rtTA mice were fed doxycycline chow (625 mg/kg, Envigo) for 28 days to induce recombination. Then all mice were switched to placebo diet for 3 days (starting day –3). On the third day (now day 0), some mice were infected intranasally with MHV68 (+MHV68 group) or dosed with saline solution (naive group) and then followed for 28 days, when their lungs were harvested for analysis. Finally, interventions were scheduled as preventive (sGC agonist diets were presented on day –3, prior to infection, to the corresponding groups) or therapeutic (sGC agonist diets were presented on day 7 postinfection to the corresponding groups).

A scheme of all interventions and endpoints is summarized in Supplemental Figure 3.

### Analysis of murine tissue

#### Histopathology.

After sacrifice, lungs were perfused with 10% neutral-buffered formalin followed by paraffin embedding. Histopathologic changes and fibrosis were evaluated using Masson’s trichrome staining (MilliporeSigma, HT15) according to manufacturer’s protocol.

#### Viral load.

Copurification of RNA and DNA was performed in lung tissue using All Prep DNA/RNA kit (QIAGEN, 80204) and genomic DNA extraction in spleen samples was completed using DNeasy isolation kit (QIAGEN, 69504) according to the manufacturer’s recommendations. Viral load of the MHV68 *Orf50* copies normalized to host *Gapdh* was performed as previously described (9).

#### Quantitative PCR analysis.

RNA from flash-frozen and pulverized lung samples was extracted using the RNeasy extraction kit (QIAGEN, 74104) following the manufacturer’s protocol. Relative gene expression was obtained using 2^-ΔΔCt^ method under housekeeping gene normalization (*Rn18s* RNA). The primers used for the real-time quantitative PCR can be found in Supplemental Table 2.

#### Hydroxyproline assay.

Frozen pulverized lung tissue samples (~5 mg per sample) were resuspended in the recommended volume of 6N HCl, then hydrolyzed at high temperature. Assay reactions were performed following the manufacturer’s protocol (MilliporeSigma, MAK008). Total amount of protein was measured, and results were expressed as per milligram of lung tissue.

#### GSH and GSSG determination.

Frozen pulverized lung tissue samples were homogenized in 100 μL of 150 mM HEPES buffer containing 50 mM NaCl pH 7.4 supplemented with 25 mM *N*-ethylmaleimide (NEM) and spiked with 1 μM ^13^C_2_^15^N-labeled GSH. After 30 minutes at 37°C, 500 μL of cold ethanol was added to the homogenate to precipitate proteins, followed by centrifugation (21,000*g*, 4°C, 10 minutes), and the supernatant was dried under an N_2_ stream at room temperature. Samples were reconstituted in water (MS grade) for liquid chromatography-tandem mass spectrometry quantification.

#### HPLC-MS.

Analyte quantification was performed using a QTRAP 6500 triple-quadrupole mass spectrometer (Sciex) equipped with an electrospray ionization source. Internal standard curves using synthetic GSH-NEM and ^3^C_2_^15^N GSH-NEM were prepared to quantify endogenous tissue and cellular GSH and GSSG levels. The following transitions in positive ion mode were used: GS-NEM (433.0/304.0), ^3^C_2_^15^N GS-NEM (436.0/307.0), and GSSG (613.2/355.2). The following settings for the mass spectrometer were used: source temperature 600°C; ionization spray voltage 5,500 V; CAD medium arbitrary units; curtain gas 40 arbitrary units; GS1 60 arbitrary units; GS2 45 arbitrary units; entrance potential 10 V; collision cell exit potential 8 V; declustering potential 70 V; collision energy 30 V. The samples were resolved on a Phenomenex C18 Omega column (2.0 × 150 mm, 3 μm particle size) using the following solvent system A) water + 0.1% formic acid and B) 0.1% formic acid in acetonitrile at a flow rate of 350 μL/min. Chromatographic conditions were as follows: 1% solvent B for 0.1 minute, followed by a linear gradient to 20% solvent B at 6 minutes, then switch to 100% solvent B for 2 minutes and re-equilibration to return to the initial condition (1% solvent B) for 4 minutes.

### Primary cell isolation

#### From murine lungs.

Primary murine AECIIs and fibroblasts were obtained from 3-month-old *Cyb5r3^fl/fl^* and *Cyb5r3* SPC–KO mice (1 mouse per isolation and 3 mice per group), and primary murine fibroblasts were obtained from 3-month-old C57BL/6J mice (1 mouse per isolation and 2 mice in total), as previously described (9). Briefly, primary cells were obtained by digesting the lungs using an enzymatic solution containing dispase (Invitrogen, 17105041), collagenase type IV (Roche, 10269638001), and DNase I (MilliporeSigma, DN25), in a Gentle MACS (Miltenyi Biotec) dissociator. MACS beads and columns (Miltenyi Biotec) were used to obtain cell suspensions by negative selection of CD45^–^ and CD31^–^ and positive selection of CD326^+^ (EpCAM) cells. CD45^–^CD31^–^CD326^+^ cells from *Cyb5r3^fl/fl^* and *Cyb5r3* SPC–KO mice were pelleted and used to confirm deletion of *Cyb5r3* in an enriched isolation of AECIIs. To obtain primary mouse lung fibroblasts, CD45^–^CD31^–^CD326^–^ cells (negative fraction from the CD326 selection) were plated in 10 cm Petri dishes and incubated in Ham’s F12 medium (Gibco) supplemented with 10% FBS (Gibco) and 1% penicillin/streptomycin (Gibco) for 2 passages. Cells were cultured at 37°C, in 5% CO_2_.

#### From human lung tissue.

Primary normal (from donor young and old) and IPF fibroblasts were isolated as previously described (9), cultured in Ham’s F12 medium (Gibco) with 10% FBS (Gibco) and 1% of antimycotic-antibiotic (Gibco), and used in passages 4 to 6. Cells were cultured at 37°C, in 5% CO_2_.

### Cell culture and treatments

#### Cell culture maintenance.

Immortalized mouse lung epithelial cells (MLE12) were obtained from ATCC (CRL-2110) and cultured in HITES media (Gibco) supplemented with 2% FBS (Gibco), 50 U/mL penicillin, and 50 μg/mL streptomycin (Gibco) in 5% CO_2_ at 37°C.

#### CYB5R3 KD.

MLE12 cells were seeded and grown to 50% confluence, then treated with nontargeted shRNA adenovirus (or *Cyb5r3*-Scr) or shRNA adenovirus targeting *Cyb5r3* (or *Cyb5r3*-KD). Cells were harvest 72 hours posttransduction. Adenovirus-infected cells (*Cyb5r3*-Scr or *Cyb5r3*-KD MLE12 cells) undergoing further stimulations received treatment 24 hours posttransduction and incubated an additional 48 hours prior to harvest.

#### Cell treatments.

Cells were treated (24 hours after adenovirus infection) with recombinant human TGF-β1 (Bio-Techne) at 5 ng/mL for time points ranging from 15 to 120 minutes and lysed in RIPA buffer for Western blotting. For cotreatment experiments cells were treated with 5 ng/mL recombinant human TGF-β1 or PBS and/or 1 μM Bay58 (Bayer), 1 μM Bay41 (Bayer), or 100 μM 8Br-cGMP (MilliporeSigma, B1381) for 48 hours. In all cases, the vehicle used was 0.01% DMSO in the HITES media. Cells were washed in PBS and lysed in TRIzol (Thermo Fisher Scientific) for RNA isolation.

#### Conditioned medium experiment.

*Cyb5r3*-Scr and *Cyr5r3*-KD MLE12 cells were cultured as described above, until recombinant TGF-β1 or PBS was added to the cell culture medium at 10 ng/mL for 8 hours. Cells in all groups were then extensively washed with plain HITES media and cultured in fresh TGF-β1–free culture medium for 48 hours. After 48 hours, the media were collected, and the MLE12 cells were washed and pelleted for RNA extraction. This 48-hour conditioned medium was used to culture primary murine fibroblasts for 24 hours (in a 1:1 ratio, conditioned medium to fresh fibroblast culture media), after which stimulatory potential was assessed using light microscopy (EVOS M7000, Thermo Fisher Scientific) observation and by quantitative PCR analysis of the lysed fibroblasts.

### Protein expression by immunoblot analysis

Cells were lysed on ice in RIPA buffer with a protease and phosphatase inhibitor mixture (MilliporeSigma, P8340 & P5726), and immunoblots were performed as previously described (9). Primary antibodies used in this study were CYB5R3 (1:1,000; Proteintech, 10894-1-AP), β-actin (1:1,000; Cell Signaling Technology, 5125), Smad 2/3 (1:1,000; Cell Signaling Technology, 8685), p-Smad 2/3 (1:1,000; Cell Signaling Technology, 8828), ERK1/2 (1:1,000; Cell Signaling Technology, 4695), p-ERK1/2 (1:1,000; Cell Signaling Technology, 4370), caspase-3 (1:1,000; Cell Signaling Technology, 14220), cleaved caspase-3 (1:1,000; Cell Signaling Technology, 9661), and vimentin (1:1,000; Cell Signaling Technology, 5741). Detection and housekeeping antibodies used in this study are listed here: HRP-conjugated β-actin (1:30,000; Proteintech, HRP-66009), HRP-conjugated mouse anti-goat (1:2,000; Santa Cruz Biotechnology, sc-2354), and HRP-conjugated mouse anti-rabbit (1:2,000; Santa Cruz Biotechnology, sc-2357).

### ELISA measurements

Active and total TGF-β1 were measured by ELISA (Invitrogen, B88-8350-22) in 100 μL of cell culture media, according to the manufacturer’s protocol. Osteopontin was measured by Mouse Osteopontin ELISA Kit (MilliporeSigma, RAB0437) in cell culture media (5-fold dilution), according to the manufacturer’s protocol.

### Statistics

All data were analyzed using GraphPad Prism, version 9.1.0 (GraphPad Software). Statistical significance of differences was evaluated by unpaired, 2-tailed Student’s *t* test or 1-way or 2-way ANOVA followed by a post hoc Tukey’s multiple-comparison test. Differences between weight loss curves were calculated using 1-way repeated measures ANOVA, and Kaplan-Meier survival curves were compared with the Mantel-Cox test. Differences were considered statistically significant at *P* < 0.05. The type of analysis used for each data set, plus the specific *n* for each experimental set, are indicated in the legends of the corresponding figures.

### Study approval

Human lung tissue was collected under University of Pittsburgh Institutional Review Board–approved and Committee for Oversight of Research and Clinical Training Involving Decedents–approved (CORID-approved) protocols (970946, PRO14010265, and CORID No. 300). All animal procurement and procedures were reviewed and approved by the Institutional Animal Care and Use Committee of the University of Pittsburgh (1911626) and adhered to the NIH *Guide for the Care and Use of Laboratory Animals* (National Academies Press, 2011).

## Author contributions

MB, ACS, and ALM conceived and designed the experiments and supervised the project. MB performed all mouse infections. MB, JC, CB, AN, AM, and BGM performed the mouse experiments. TK carried out immunofluorescence staining and ELISAs. MB and MPM performed cell culture experiments. SRS and FJS performed and analyzed the HPLC-MS data. MB, JC, TK, MPM, CB, AN, DA, LR, JB, SS, AM, NU, BGM, and KF carried out experiments and analyzed experimental data. SAH, BGM, and MR performed the required mouse surgeries. JS procured and processed patient samples. MB, JC, TK, MPM, ACS, and ALM interpreted results of experiments. MB prepared figures. TK and MB wrote the manuscript. MB, TK, JC, MPM, PS, ACS, and ALM edited the manuscript. All authors approved the final version of the manuscript for submission.

## Supplementary Material

Supplemental data

## Version 1 02/07/2023

In-Press Preview

## Version 2 03/08/2023

Electronic publication
