# Supplementary material for: CYB5R3 in type II alveolar epithelial cells protects against lung fibrosis by suppressing TGF-β1 signaling
Source: JCI Insight. 2023 Mar 8;8(5):e161487. doi: 10.1172/jci.insight.161487 (PMC10077481; doi:10.1172/jci.insight.161487)
Supplement: Supplemental data [file jciinsight-8-161487-s186.pdf]

Supplemental material

Supplemental Figure 1. Levels of NAD+ consuming and generating enzymes in different compartments of the fibrotic lung.

Supplemental Figure 2. Confirmation of *Cyb5r3* deletion in murine AECIIs.

Supplementary Figure 3. Schematics of the in vivo experiments performed.

Supplemental Figure 4. Reproducibility of the MHV68-induced lung fibrosis model in *Cyb5r3* SPC-KO mice.

Supplemental Figure 5. Other markers of fibrosis, inflammation, and injury in the MHV68-induced lung fibrosis model in *Cyb5r3* SPC-KO mice.

Supplemental Figure 6. Increased expression of markers of fibrosis and senescence in the lung of the *Cyb5r3* SPC-KO mice after bleomycin.

Supplemental Figure 7. Mitochondrial intervention does not improve virus-induced lung fibrosis in *Cyb5r3* SPC-KO mice.

Supplemental Figure 8. Effects of a short-term therapeutic intervention with sGC activator BAY 54-6544 in lung and liver of *Cyb5r3* SPC-KO infected mice.

Supplemental Figure 9. Preventive intervention with sGC activator ameliorates fibrotic endpoints in *Cyb5r3* SPC-KO mice after MHV68-induced lung fibrosis at day 28.

Supplemental Figure 10. Therapeutic intervention with sGC agonists in the absence of injury.

Supplemental Figure 11. Therapeutic intervention with sGC agonists ameliorates fibrotic and senescence outcomes in *Cyb5r3* SPC-KO mice after injury at 28 days.

Supplemental Figure 12. CYB5R3 deficiency is associated with oxidative stress and a pro-fibrotic phenotype.

Supplemental Figure 13. CYB5R3 modulates expression of TGF-β1-target genes via sGC-dependent pathways.

Supplemental Figure 14. Effects of sGC agonists on primary mouse lung fibroblasts in culture.

Supplemental Table 1. Demographic characteristics of the patients cohort.

Supplemental Table 2. List of PrimeTime® primer assays (Integrated DNA Technologies)

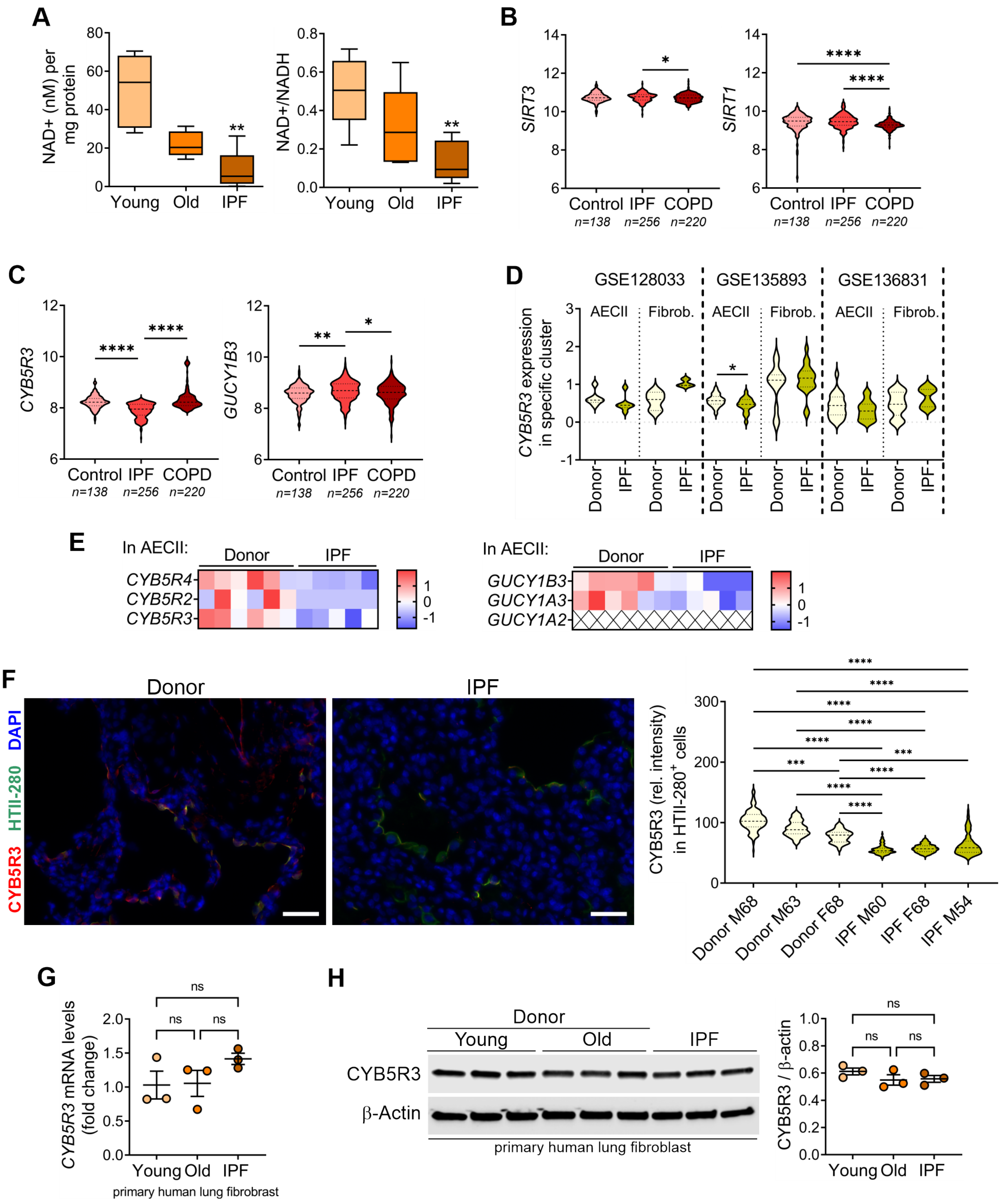

Supplemental Figure 1. Levels of NAD+ consuming and generating enzymes in different compartments of the fibrotic lung. (**A**) Levels of normalized NAD$^+$ and NAD$^+$/NADH ratio are reduced with age and in IPF lung compared with young donors

(Min-to-max with median, *n* = 5/group). Relative gene expression levels of *SIRT3* and *SIRT1* (**B**) and *CYB5R3* and *GUCY1B3* (**C**) on a large mRNA bulk expression dataset containing ILD and COPD patients and healthy controls from the Lung Tissue Research Consortium (Violin plots with median and quartiles; *n* = 91 donor / 194 ILD / 144 COPD in GSE47460). See reference (24) (**D**) Mean *CYB5R3* expression in AECII and fibroblast clusters from three different scRNA-seq studies comparing donor and IPF lung. (Violin plots with median and quartiles; *n* = 6 donor / 7 IPF in GSE128033; *n* = 32 donor / 28 IPF in GSE135893; *n* = 18 donor / 10 IPF in GSE136831). See reference (63) for data compilation. (**E**) Heatmap analysis of other *CYB5R* and *GUCY1* isoforms in AECII in scRNA-seq data from donor and IPF patients (GSE128033). Color scale denotes Z-score of the normalized fold change expression. (**F**) Different representative immunofluorescence using anti HTII-280 (AECII marker; green) and anti–CYB5R3 (red) antibodies (*n* = 3/group). Scale bars: 50 μm. Individual quantification of the different samples of CYB5R3 staining (as relative pixel intensity) of individual AECII (HTII-280+) cells per condition. (Violin plots with median and quartiles). CYB5R3 transcript (**G**) and protein levels (**H**) in primary lung fibroblasts isolated from young and old donors, and IPF lungs (Individual data with mean ± SEM, *n* = 3/group). Statistical analysis was performed using 1-way ANOVA with multiple comparison test (A-C, F-G) and unpaired, 2-tailed Student's t test (D); ns, no significant; *p<0.05, **p<0.01, ***p<0.001, ****p<0.0001.

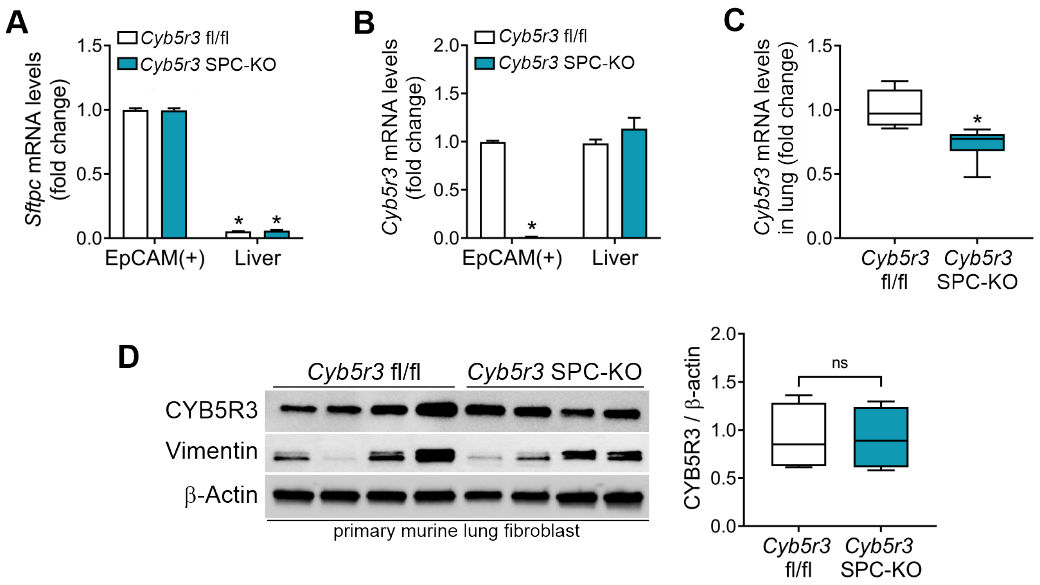

Supplemental Figure 2. Confirmation of *Cyb5r3* deletion in murine AECIIs. (**A**) Enrichment of AECII from *Cyb5r3* fl/fl and *Cyb5r3* SPC-KO mice lungs was performed and levels of *Sftpc* were tested. (Data are reported as mean ± SEM, *n* = 3/group). (**B**) In the enriched fraction, cells derived from *Cyb5r3* SPC-KO lungs did not express *Cyb5r3* transcript at a detectable level. (Data are reported as mean ± SEM, *n* = 3/group). (**C**) Total lung lysate from conditional type II lung epithelial cells *Cyb5r3* knockout mice show less expression of *Cyb5r3* mRNA. (Min-to-max with median, *n* = 6/group). (**D**) Representative immunoblot and quantification of CYB5R3 transcript in primary lung fibroblasts isolated from *Cyb5r3* fl/fl and *Cyb5r3* SPC-KO mice lungs. Using vimentin as fibroblast fraction marker. (Min-to-max with median, *n* = 4/group).

Statistical analysis was performed using 2-way ANOVA with multiple comparison test (A-B), 2-tailed Student's t test (C-D); *$p<0.05$; n.s., non-significant.

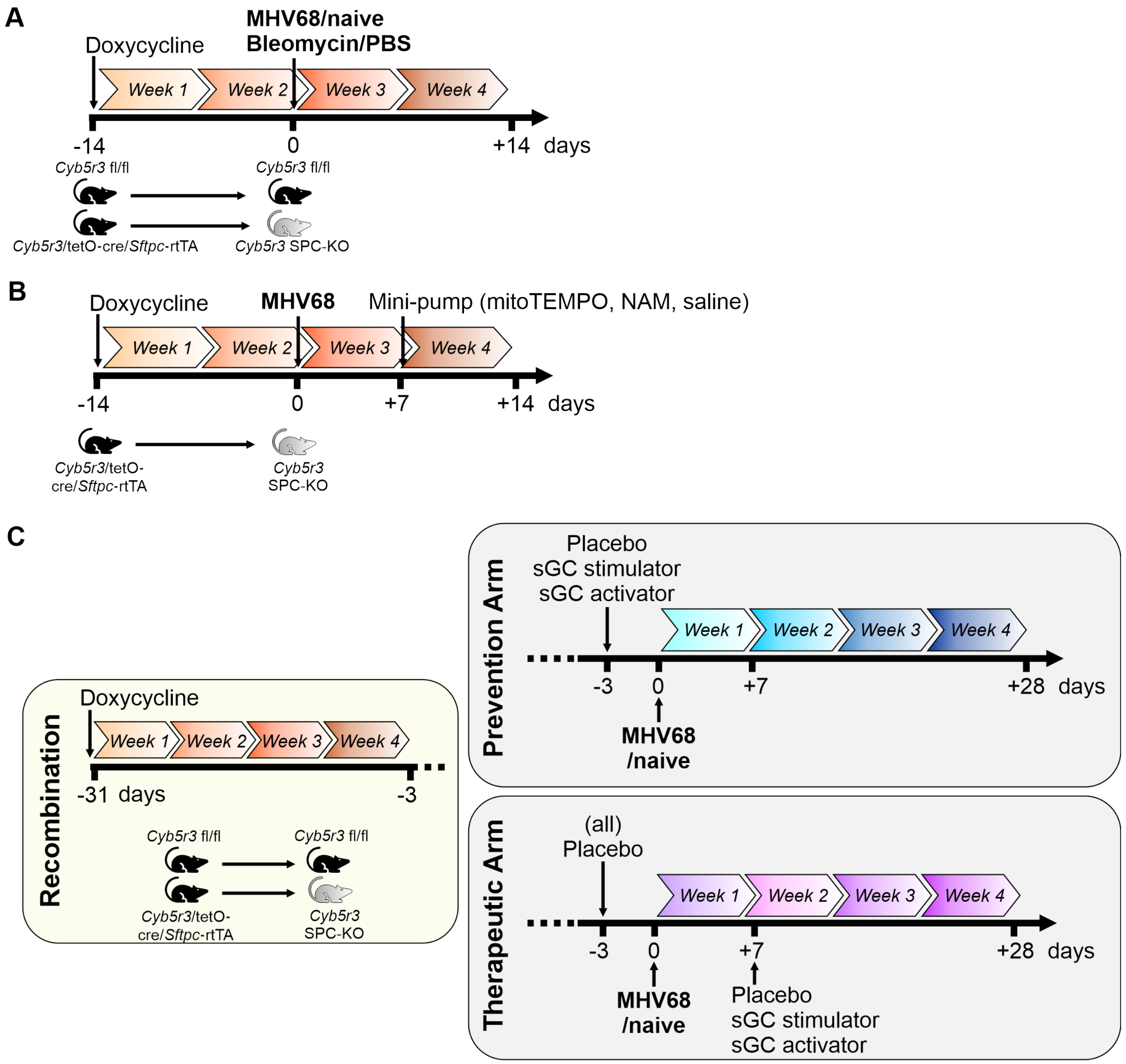

Supplementary Figure 3. Schematics of the in vivo experiments performed. (**A**) MHV68 and bleomycin models of lung injury and fibrosis. (**B**) Mitochondrial therapeutic intervention in MHV68 model. (**C**) Preventive and therapeutic interventions using sGC agonists (sGC stimulator BAY 41-8543 or sCG activator 54-6544) in MHV68 model.

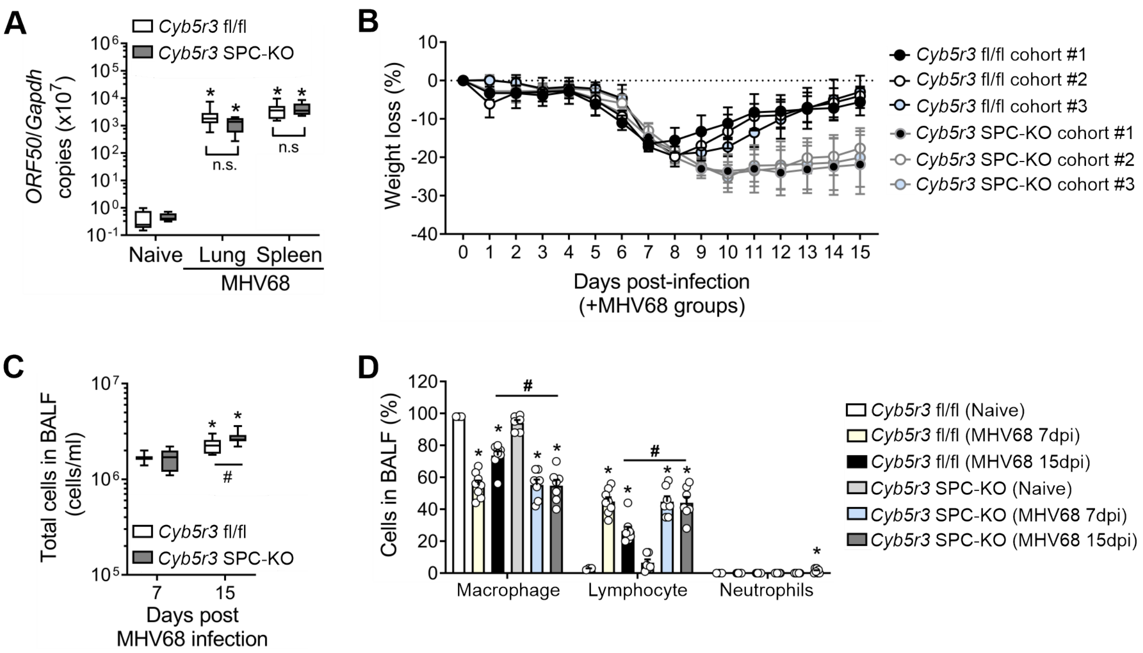

Supplemental Figure 4. Reproducibility of the MHV68-induced lung fibrosis model in *Cyb5r3* SPC-KO mice. (**A**) Viral load by qPCR from *Cyb5r3* fl/fl and *Cyb5r3* SPC-KO mice at 15 days post-infection. (Min-to-max with median, *n* = 6-8/group). (**B**) Weight loss data in *Cyb5r3* fl/fl and *Cyb5r3* SPC-KO mice after MHV68 infection performed as three different cohorts (started on different dates at different locations in the vivarium). (Data point is mean ± SD, *n* = 6). Total cell numbers (**C**) and differential cells populations (**D**) in the BALF (bronchoalveolar lavage fluid) at different time points after MHV68 infection. (C: Min-to-max with median, *n* = 6-8/group; D: Individual values with mean ± SEM, *n* = 6-8/group). Statistical analysis was performed using 2-way ANOVA with multiple comparison test (A, C-D), and 1-way repeated measures ANOVA (B); versus naïve: *p<0.05; as indicated: #p<0.05; n.s., non-significant.

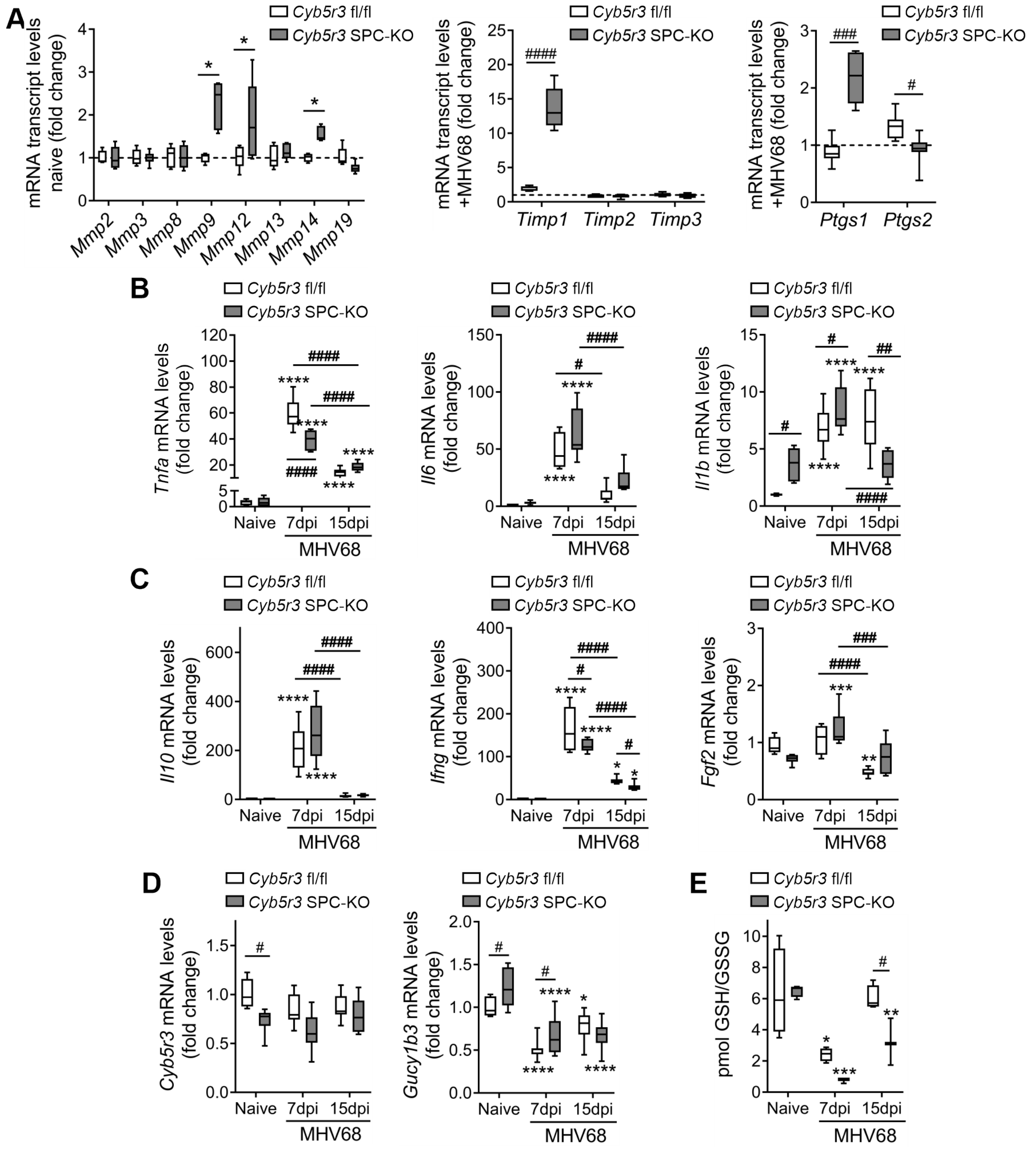

Supplemental Figure 5. Other markers of fibrosis, inflammation, and injury in the MHV68-induced lung fibrosis model in *Cyb5r3* SPC-KO mice. (**A**) Changes in the relative transcript levels of different *Mmps* in the naïve lung. Relative changes of the different *Timps* and *Ptgs* isoforms upon MHV68 infection. (Min-to-max with median, *n* = 6-8/group). (**B**) Relative expression changes of inflammation markers *Tnfa*, *Il6* and *Il1b* mRNA levels after virus infection in *Cyb5r3* fl/fl and *Cyb5r3* SPC-KO mice. (Min-to-max

with median, *n* = 6-8/group). (**C**) Relative expression changes of injury markers *Il10*, *Ifng* and *Fgf2* mRNA levels after virus infection. (Min-to-max with median, *n* = 6-8/group). (**D**) Transcript changes upon infection in *Cyb5r3* and *Gucy1b3* in *Cyb5r3* fl/fl and *Cyb5r3* SPC-KO mice. (Min-to-max with median, *n* = 6-8/group). (**E**) The ratio of reduced (GSH) and oxidized (GSSG) glutathione levels in *Cyb5r3* fl/fl and *Cyb5r3* SPC-KO mouse lungs after infection. (Min-to-max with median, *n* = 6-8/group). Statistical analysis was performed using 2-way ANOVA with multiple comparison test (A-E), and unpaired, 2-tailed Student's t test (A); versus naïve: *p<0.05, **p<0.01, ***p<0.001, ****p<0.0001; as indicated: #p<0.05, ##p<0.01, ###p<0.001, ####p<0.0001.

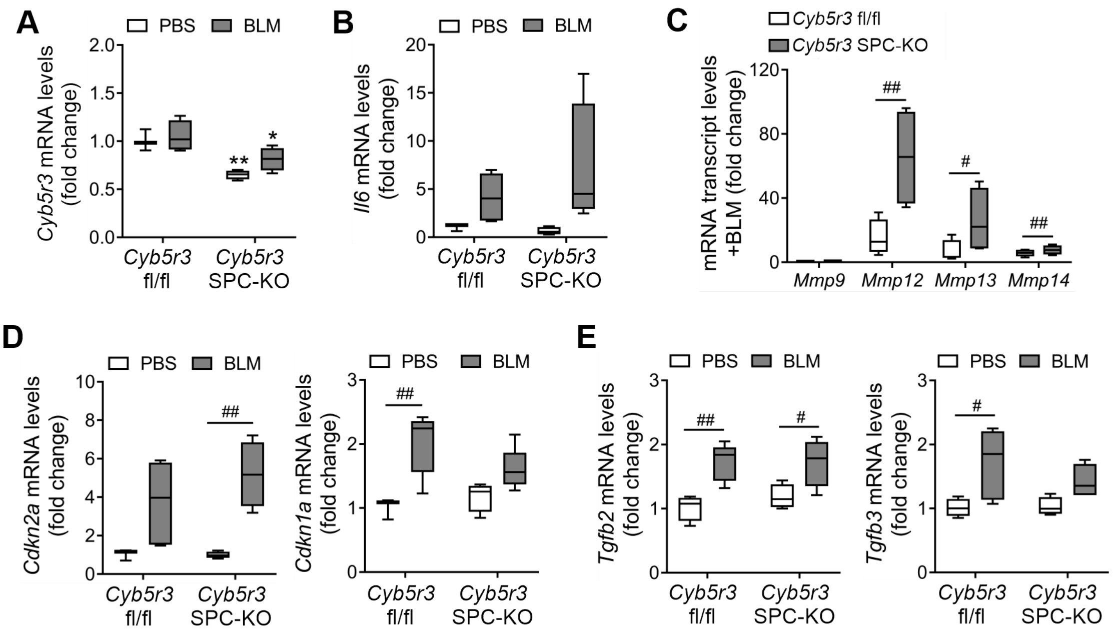

Supplemental Figure 6. Increased expression of markers of fibrosis and senescence in the lung of the *Cyb5r3* SPC-KO mice after bleomycin. (**A**) *Cyb5r3* transcript levels after bleomycin in total lung lysate. (Min-to-max with median, *n* = 4-5/group). (**B**) Relative expression changes of SASP markers *Il6* mRNA levels after bleomycin in *Cyb5r3* fl/fl and *Cyb5r3* SPC-KO mice. (Min-to-max with median, *n* = 4-5/group). (**C**) Relative expression of different *Mmps* transcripts after bleomycin treatment in *Cyb5r3* fl/fl and *Cyb5r3* SPC-KO mice. (Min-to-max with median, *n* = 4-5/group). (**D**) Relative expression of senescence associated transcripts *Cdkn2a* and *Cdkn1a* after bleomycin treatment in *Cyb5r3* fl/fl and *Cyb5r3* SPC-KO mice. (Min-to-max with median, *n* = 4-5/group). (**E**) Relative expression of *Tgfb2* and *Tgfb3* transcripts after bleomycin treatment in *Cyb5r3* fl/fl and *Cyb5r3* SPC-KO mice. (Min-to-max with median, *n* = 4-5/group). Statistical analysis was performed using 2-way ANOVA with multiple comparison test (A-B, D-E) and unpaired, 2-tailed Student's t test (C); versus PBS: *p<0.05, **p<0.01; as indicated: #p<0.05, ##p<0.01.

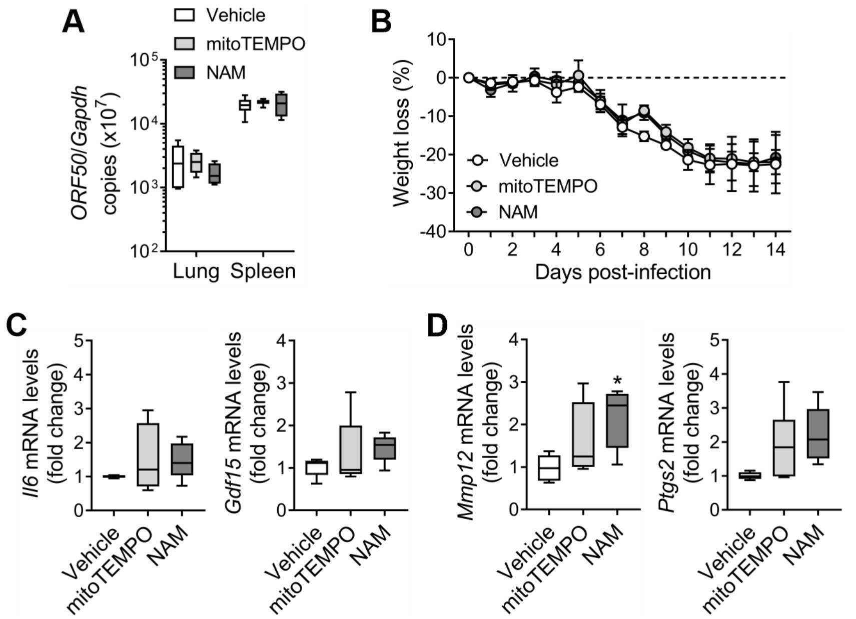

Supplemental Figure 7. Mitochondrial intervention does not improve virus-induced lung fibrosis in *Cyb5r3* SPC-KO mice. (**A**) Viral load by qPCR from the differently treated *Cyb5r3* SPC-KO mice at 15 days post-infection. (Min-to-max with median, *n* = 5-6/group). (**B**) Weight loss data of the three different treatment groups followed for 15 days post infection. Osmotic pump implantation was performed on day 7 after infection. (Data point is mean ± SD, *n* = 6). (**C**) No detectable change of *Il6* and *Gdf15* transcript levels after treatments in *Cyb5r3* SPC-KO MHV68-infected mice. (Min-to-max with median, *n* = 5-6/group). (**D**) Relative expression changes of profibrotic markers *Mmp12* and *Ptgs2* mRNA levels after treatments in *Cyb5r3* SPC-KO MHV68-infected mice. (Min-to-max with median, *n* = 5-6/group). Statistical analysis was performed using 1-way ANOVA with multiple comparison test (A, C-D) and 1-way repeated measures ANOVA (B); versus vehicle: *p<0.05.

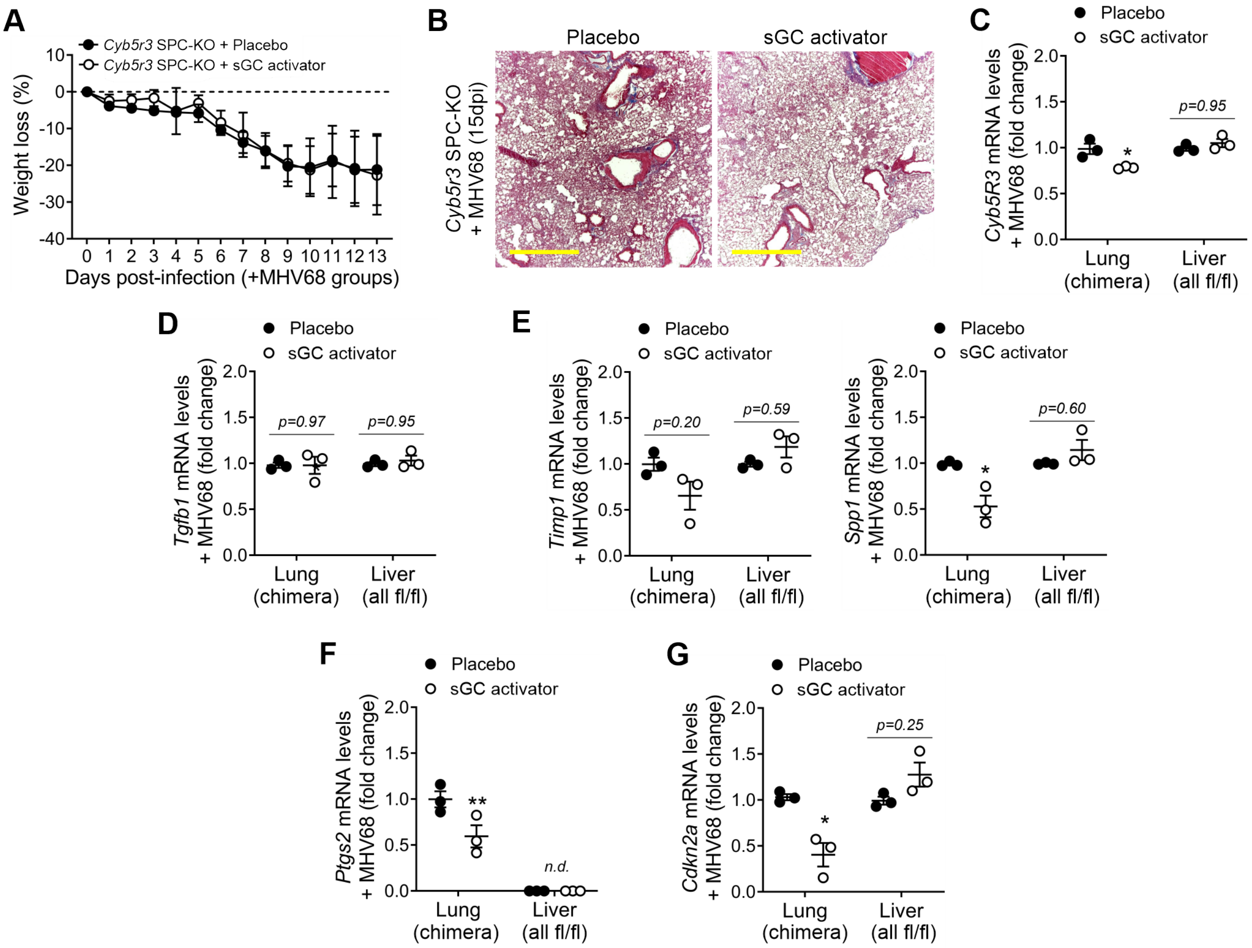

Supplemental Figure 8. Effects of a short-term therapeutic intervention with sGC activator BAY 54-6544 in lung and liver of *Cyb5r3* SPC-KO infected mice. (**A**) Weight loss data comparing AECII CYB5R3 deficient mice after infection in the different treatment arms (Placebo vs. sGC activator BAY 54-6544). (Data point is mean ± SD, *n* = 3/group). (**B**) Representative Masson trichrome staining in lung sections *Cyb5r3* SPC-KO mice at day 14 post MHV68 infection with different intervention. Scale bars: 500 µm. (*n* = 3/group). Change in transcript levels of *Cyb5r3* (**C**) and *Tgfb1* (**D**) after treatment in *Cyb5r3* SPC-KO MHV68-infected mice. (Individual data with mean ± SEM, *n* = 3/group). (**E**) Levels of mRNA of *Timp1* and *Spp1* after treatment in lung and liver of *Cyb5r3* SPC-KO MHV68-infected mice. (Individual data with mean ± SEM, *n* = 3/group). Change in transcript levels

of *Ptgs2* (**F**) and *Cdkn2a* (**G**) after treatment in *Cyb5r3* SPC-KO MHV68-infected mice. (Individual data with mean ± SEM, *n* = 3/group). Statistical analysis was performed using 1-way repeated measures ANOVA (A), and 2-way ANOVA with multiple comparison test (C-G); as indicated: *p<0.05, **p<0.01; n.d., non-detected. Chimera: total lung lysate will be a mixture of KO and WT (fl/fl) cells.

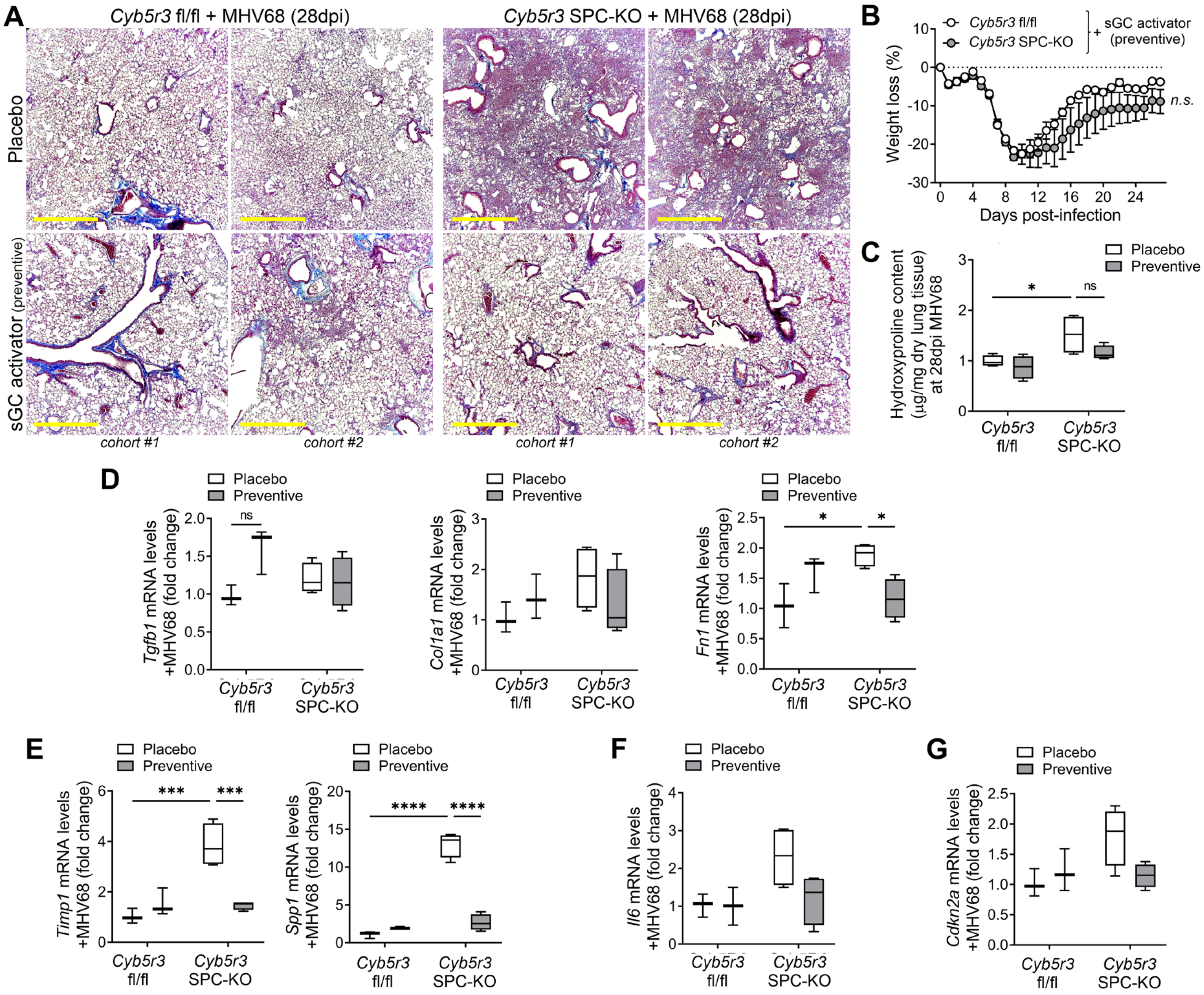

Supplemental Figure 9. Preventive intervention with sGC activator ameliorates fibrotic endpoints in *Cyb5r3* SPC-KO mice after MHV68-induced lung fibrosis at day 28. (**A**) Representative Masson trichrome staining in lung sections from *Cyb5r3* fl/fl and *Cyb5r3* SPC-KO mice at day 28 post MHV68 infection with different interventions representing different experimental cohorts (Placebo vs. sGC activator BAY 54-6544 diet). All interventions were administered mixed in the same base chow composition as the Placebo diet and started 3 days before the MHV68 infection took place. Scale bars: 500 µm. (*n* = 4-6/group) (**B**) Weight loss data comparing fl/fl and AECII CYB5R3 deficient mice after infection in the activator preventive treatment arm. (Data point is mean ± SD,

n= 4-6). (**C**) Collagen deposition in lungs of *Cyb5R3* fl/fl and *Cyb5R3* SPC-KO infected mice after the preventive intervention with sGC activator determined by hydroxyproline levels. (Min-to-max with median, *n* = 4-6/group). (**D**) Relative expression changes of fibrotic markers *Tgfb1*, *Col1a1* and *Fn1* mRNA levels after preventive treatment in *Cyb5R3* fl/fl and *Cyb5R3* SPC-KO MHV68-infected mice. (Min-to-max with median, *n* = 4-6/group). (**E**) Change in transcript levels of *Timp1* and *Spp1* after treatments in *Cyb5R3* fl/fl and *Cyb5R3* SPC-KO MHV68-infected mice after preventive treatment. (Min-to-max with median, *n* = 4-6/group). Change in transcript levels of *Il6* (**F**) and *Cdkn2a* (**G**) after preventive intervention with sGC activator in *Cyb5R3* fl/fl and *Cyb5R3* SPC-KO MHV68-infected mice. (Min-to-max with median, *n* = 4-6/group). Statistical analysis was performed using 1-way repeated measures ANOVA (B), and 2-way ANOVA with multiple comparison test (C-G); as indicated: *$p<0.05$, ***$p<0.001$, ****$p<0.0001$; n.s., non-significant.

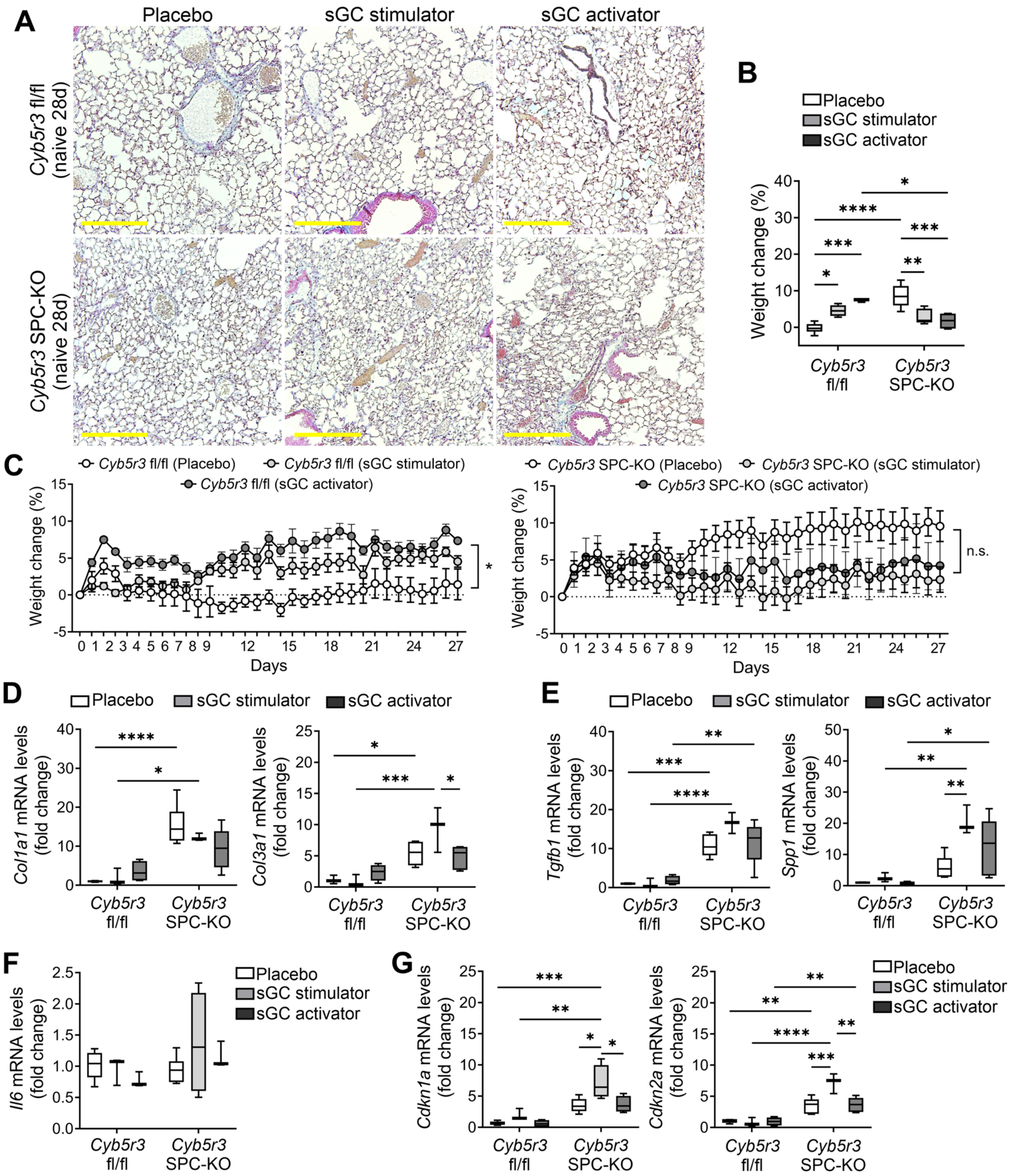

Supplemental Figure 10. Therapeutic intervention with sGC agonists in the absence of injury. (**A**) Representative Masson trichrome staining in lung sections from *Cyb5R3* fl/fl and *Cyb5R3* SPC-KO mice at day 28 with different interventions (Placebo, sGC stimulator BAY 41-8543 or sGC activator BAY 54-6544). All interventions were administered mixed in the same base chow composition as the Placebo diet and started at day 7. Scale bars: 500 µm. (*n* = 5-6/ group). (**B**) Percentage of weight change

comparing naïve fl/fl mice in the different treatment arms. (Data point is mean ± SD, *n* = 5-6). (**C**) Percentage of weight change comparing naïve *Cyb5R3* SPC-KO mice in the different treatment arms. (Data point is mean ± SD, *n*= 5-6). (**D**) Relative expression changes of fibrotic markers *Col1a1* and *Col3a1* mRNA levels after treatments in uninjured *Cyb5R3* fl/fl and *Cyb5R3* SPC-KO mice. (Min-to-max with median, *n* = 5-6/group). (**E**) Change in transcript levels of *Tgfb1* and *Spp1* after treatments in *Cyb5R3* fl/fl and *Cyb5R3* SPC-KO uninjured mice. (Min-to-max with median, *n* = 5-6/group). (**F**) Relative expression changes of *Il6* mRNA levels after treatments in uninjured *Cyb5R3* fl/fl and *Cyb5R3* SPC-KO mice. (Min-to-max with median, *n* = 5-6/group). (**G**) Change in transcript levels of *Cdkn1a* and *Cdkn2a* after treatments in *Cyb5R3* fl/fl and *Cyb5R3* SPC-KO uninjured mice. (Min-to-max with median, *n* = 5-6/group). Statistical analysis was performed using 2-way ANOVA with multiple comparison test (B, D-G) and 1-way repeated measures ANOVA (C); as indicated: *p<0.05, **p<0.01, ***p<0.001; ****p<0.0001; n.s., non-significant.

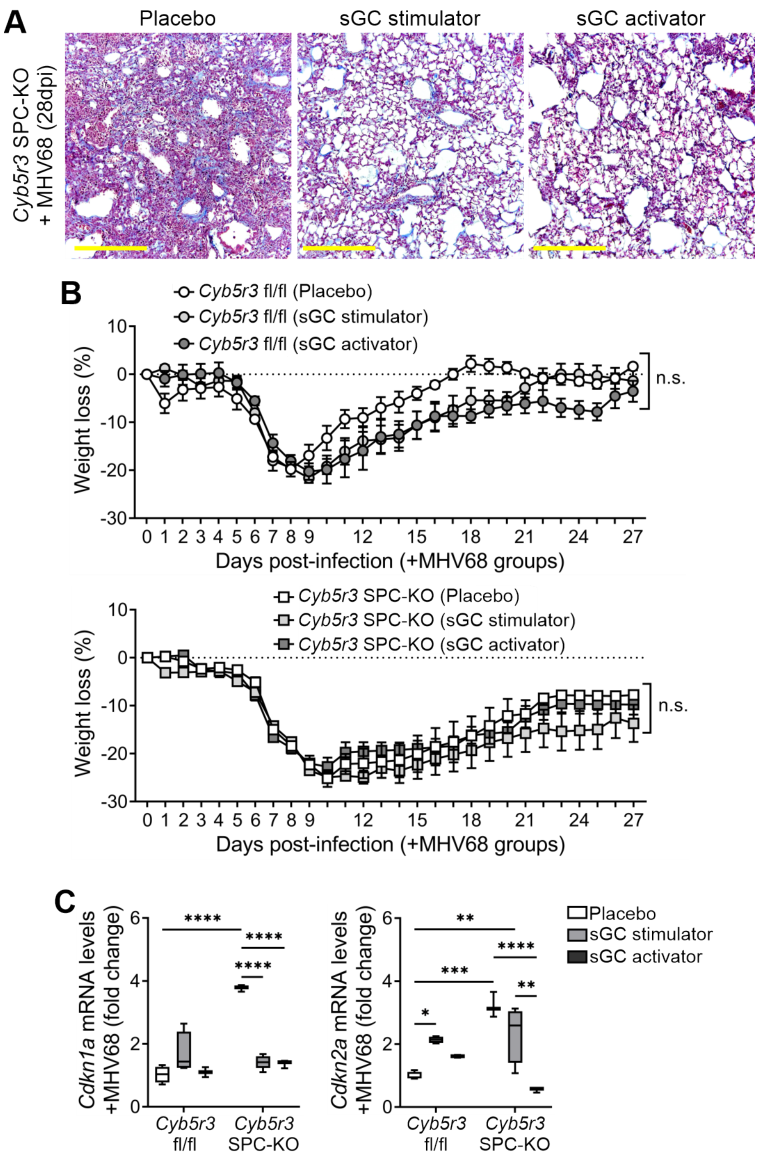

Supplemental Figure 11. Therapeutic intervention with sGC agonists ameliorates fibrotic and senescence outcomes in *Cyb5r3* SPC-KO mice after injury at 28 days. (**A**) Representative Masson trichrome staining in lung sections from *Cyb5R3* fl/fl and *Cyb5R3* SPC-KO mice at day 28 post MHV68 infection with different interventions (Placebo, sGC stimulator BAY 41-8543 or sGC activator BAY 54-6544). Scale bars: 200 µm. (*n* = 12-4/group) (**B**) Weight loss data comparing treatments in the presence or

absence of CYB5R3 in the AECII. (Data point is mean ± SD, *n* = 4-12). (**C**) Change in transcript levels of *Cdkn1a* and *Cdkn2a* after treatments in *Cyb5R3* fl/fl and *Cyb5R3* SPC-KO MHV68-infected mice. (Min-to-max with median, *n* = 4-12/group). Statistical analysis was performed using 1-way repeated measure ANOVA (B), and 2-way ANOVA with multiple comparison test (C); as indicated: *p<0.05, **p<0.01, ***p<0.001; ****p<0.0001; n.s., non-significant.

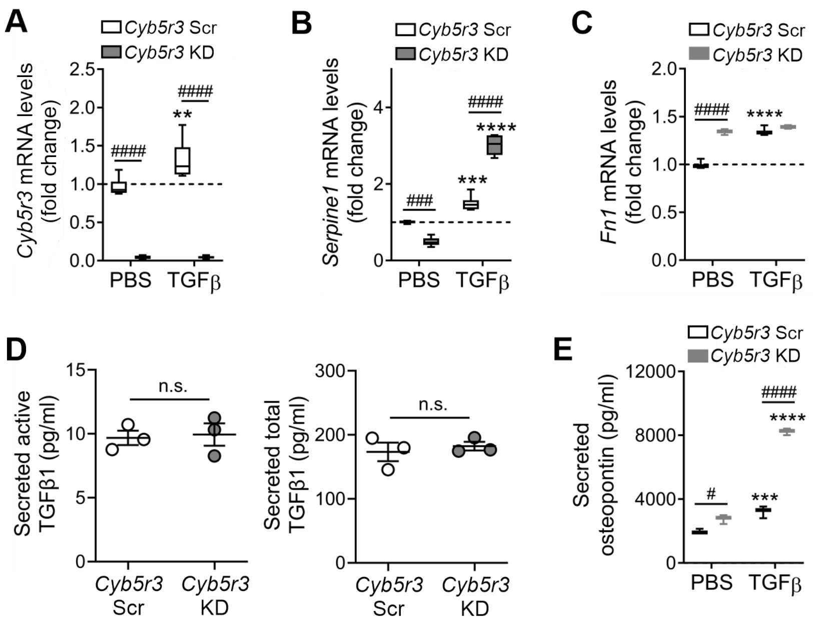

Supplemental Figure 12. CYB5R3 deficiency is associated with oxidative stress and a pro-fibrotic phenotype. Transcript levels of *Cyb5r3* (**A**), *Serpine1* (**B**) and *Fn1* (**C**) upon stimulation with TGF-β (8h stimulation, 48h wash-out), in *Cyb5r3* Scr and *Cyb5r3* KD MLE12 cells. (Min-to-max with median, *n* = 3/group). (**D**) Levels of secreted active and total TGF-β1 in the media of *Cyb5r3* Scr and *Cyb5r3* KD MLE12 cells after 48h in culture (Individual data with mean ± SEM, *n* = 3/group). (**E**) Levels of secreted osteopontin in the media of *Cyb5r3* Scr and *Cyb5r3* KD MLE12 cells after 48h in culture (Min-to-max with median, *n* = 3/group). Statistical analysis was performed using 2-way ANOVA with multiple comparison test (A-C, E) and 2-tailed Student's t test (D); versus PBS: **p<0.01, ***p<0.001, ****p<0.0001; as indicated: ###p<0.001, ####p<0.0001; n.s., non-significant.

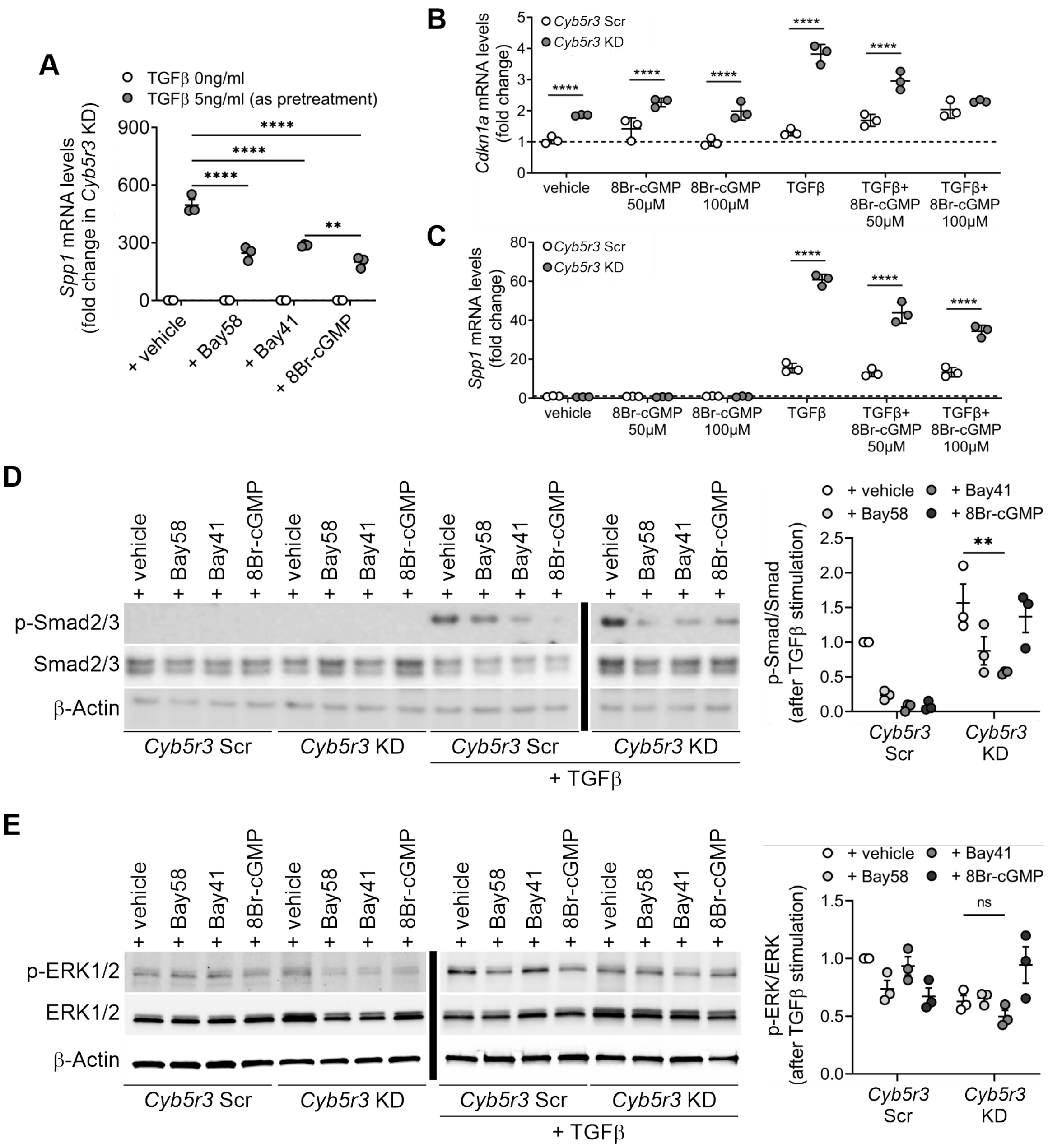

Supplemental Figure 13. CYB5R3 modulates expression of TGF-β1-target genes via sGC-dependent pathways. (**A**) Diminution of the TGFβ-mediated expression of *Spp1* in *Cyb5r3* KD MLE12 cells also if the TGF-β stimulation is performed as a pre-treatment (administered 30 min before the sGC agonist or the 8Br-cGMP). (Individual data with mean ± SEM, *n* = 3/group). The action of cGMP over the expression of the TGF-β1-target genes *Cdkn1a* (**B**) and *Spp1* (**C**) is dose dependent in *Cyb5r3* Scr and *Cyb5r3* KD MLE12

cells. (TGF-β1 stimulation: 5ng/ml for 48h; all treatments administered simultaneously) (Individual data with mean ± SEM, *n* = 3/group). (**D**) Representative immunoblot and quantification of the phosphorylation of Smad2/3 in *Cyb5r3* Scr and *Cyb5r3* KD MLE12 cells stimulated with 5 ng/ml of TGF-β1 for 30min in the presence of different sGC agonists (pre-treatments for 30min before stimulation). (Individual data with mean ± SEM, *n* = 3/group). (Black bar separate data from two separate blots). (**E**) Representative immunoblot and quantification of the phosphorylation of ERK1/2 in *Cyb5r3* Scr and *Cyb5r3* KD MLE12 cells stimulated with 5 ng/ml of TGF-β1 for 30min in the presence of different sGC agonists (pre-treatments for 30min before stimulation). (Individual data with mean ± SEM, *n* = 3/group). (Black bar separate data from two separate blots). Statistical analysis was performed using 1-way ANOVA with multiple comparison test (A-C), and 2-way ANOVA with multiple comparison test (D-E); as indicated: *p<0.05; **p<0.01; ***p<0.0001; ****p<0.00001.

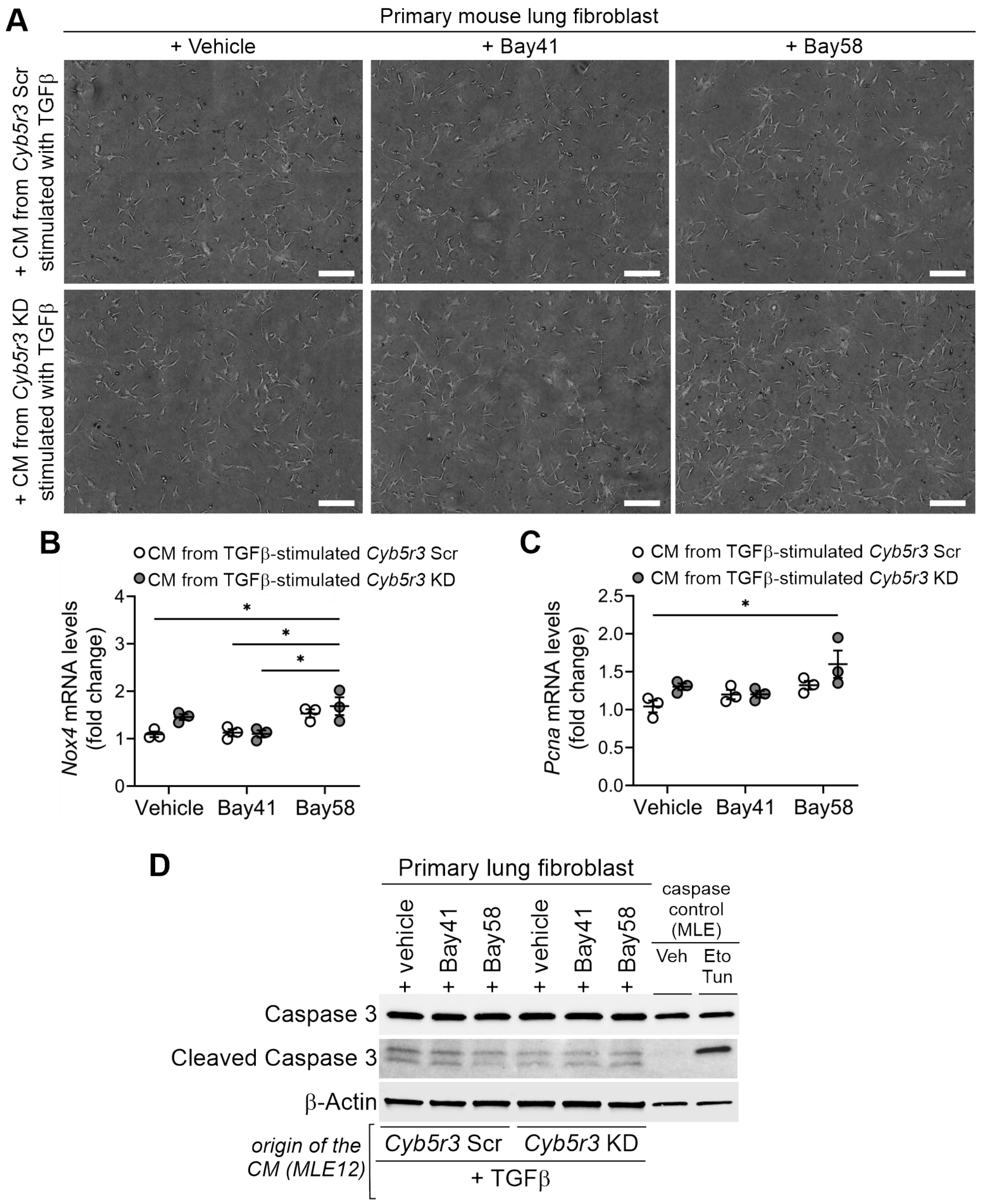

Supplemental Figure 14. Effects of sGC agonists on primary mouse lung fibroblasts in culture. (**A**) Representative images of murine primary lung fibroblasts cultured for 48h in the presence of conditioned media (CM) derived from *Cyb5r3* Scr or *Cyb5r3* KD MLE12 cells previously stimulated with TGF-β in the presence/absence of different sGC agonists (BAY 58-2667 1 μM or BAY 41-2272 1 μM). Fibroblasts were preincubated in vehicle (0.01% DMSO) or sGC agonist for 30 min before receiving the CM (in a 1:1 ratio, CM to fresh fibroblast culture media). To generate the CM media, at

24h post adenovirus-infection, *Cyb5r3* Scr or *Cyb5r3* KD MLE12 cells received recombinant TGF-β1 or PBS at 10 ng/ml for 8 hours, then (after extensive washes) cells were cultured in fresh TGF-β1-free culture media for 24 hours and media were harvested. Scale bars: 275 μm. (*n* = 3/condition). (**B**) Levels of *Nox4* and *Pcna* mRNA transcript from primary fibroblast cultured in conditioned media derived from *Cyb5r3* Scr or *Cyb5r3* KD MLE12 cells previously stimulated with TGFβ in the presence of sGC modulators as described above. (Individual data with mean ± SEM, *n* = 3/group). (**C**) Representative immunoblot detecting changes in levels of cleaved caspase 3 in the same samples. (*n* = 3). Statistical analysis was performed using 2-way ANOVA with multiple comparison test (B-C); as indicated: *$p<0.05$.

Supplemental Table S1. Demographic characterictic of the patients cohort

NAD+/NADH cohort			
	**Donor (Young)**	**Donor (Old)**	**IPF**
**Subjects**	5	5	5
**Age**	27±5<br>(20 – 32)	72±7<br>(65 – 80)	68±3<br>(64 – 74)
**Female**	2 (40%)	2 (40%)	2 (40%)
**Male**	3 (60%)	3 (60%)	3 (60%)

Cyb5R3 immunofluorescence cohort		
	**Donor**	**IPF**
**Subjects**	3	3
**Age**	66±2<br>(63 – 68)	60±6<br>(54 – 68)
**Female**	1 (33%)	1 (33%)
**Male**	2 (67%)	2 (67%)

Human lung Fibroblast origin cohort			
	**Donor (Young)**	**Donor (Old)**	**IPF**
**Subjects**	3	3	3
**Age**	24±3<br>(22 – 27)	66±7<br>(62 – 74)	60±3<br>(58 – 63)
**Female**	1 (33%)	2 (66%)	2 (66%)
**Male**	2 (66%)	1 (33%)	1 (33%)

Supplemental Table 2. List of PrimeTime® primer assays (Integrated DNA Technologies)

Gene	Assay ID
Rn18s	Mm.PT45.122532.g
Tgfb1	Mm.PT.58.11254750
Sftpc	Mm.PT.58.9922071
Cdkn1a	Mm.PT.51.17125846
Cdkn2a	Mm.PT.51.5632963
Cyb5r3	Mm.PT.58.10895816
Il6	Mm.PT.49a.11799101.g
Col1a1	Mm.PT.47.12668954
Col3a1	Mm.PT.58.32580981
Fn1	Mm.PT.58.8135568
Spp1	Mm.PT.58.43709208
Timp1	Mm.PT.53A.30682575
Timp2	Mm.PT.53a.31075226
Timp3	Mm.PT.53a.5548479
Mmp2	Mm.PT.49a.10834743
Mmp3	Mm.PT.53a.10373662
Mmp8	Mm.PT.53a.11894446
Mmp9	Mm.PT.47.7474177.g
Mmp12	Mm.PT.47.17468164
Mmp13	Mm.PT.53a.9491926
Mmp14	Mm.PT.53a.5295525
Mmp19	Mm.PT.53a.6013546
Ptgs1	Mm.PT.58.13984299
Ptgs2	Mm.PT.58.9154407
Il1b	Mm.PT.49a.17212823
Il10	Mm.PT.49a.11509489.gs

Ifng	Mm.PT.58.41769240
Fgf2	Mm.PT.56a.5129235
Gucy1b3	Mm.PT.58.14181337
Gdf15	Mm.PT.58.13112185
Cycs	Mm.PT.49a.10814950.g
Ppara	Mm.PT.49a.9374886
Serpine1	Mm.PT.58.6413525
Tgfb2	Mm.PT.58.14105470
Tgfb3	Mm.PT.58.6019015
